# Next-Generation Neural Mass Models Reproduce Features of Speech Processing

**DOI:** 10.1523/ENEURO.0379-25.2026

**Published:** 2026-08-21

**Authors:** Andrew Shannon, David Andrew William Barton, Martin Homer, Conor Houghton

**Affiliations:** ^1^School of Computer Science, University of Bristol, Bristol BS8 1TH, United Kingdom; ^2^School of Engineering Mathematics and Technology, University of Bristol, Bristol BS8 1TH, United Kingdom

**Keywords:** biophysical modeling, mathematical modeling, neural mass model, speech processing, syllable segregation

## Abstract

Segregation of speech into syllables is a key step in neural speech processing. It relies on the alignment of neural activity with the rhythmic structure of speech. Two competing hypotheses explain this “neural speech tracking”, phase-resetting and evoked responses. While phenomenological modeling of these hypotheses has been successful, we still lack understanding of the underlying cortical circuits. To investigate these mechanisms, we evaluate whether a biophysical next-generation neural mass model (NMM) can reproduce several features of neural speech tracking, using phenomenological models of the competing hypotheses as algorithmic baselines. We investigate the models’ dynamics with four tests: recreating in silico an EEG experiment that identified a correlation between tracking strength and phoneme sharpness, computing the phase concentration metric, testing the effect of varying syllabic rates, and evaluating the inter event phase coherence (IEPC) across phoneme onsets. While all of the models that we study reproduce the sharpness-tuned rhythmic speech tracking, the evoked model requires a pre-processed acoustic edge impulse stimulus. We demonstrate that the NMM is performing thresholded phase-resetting triggered by sharp onsets in the continuous speech envelope. This produces cross-frequency nested oscillations that qualitatively match an experimentally-observed dual-peak signature in the IEPC. Our results indicate that the biophysical NMM provides a mechanistic bridge between generic oscillatory dynamics in cortical populations and the cognitive computations of speech tracking. Indeed, the nonlinear dynamics of the NMM offer an explanation for how peak-rate event representations in auditory cortex activity arise in response to continuous acoustic input.

## Significance Statement

Syllable segregation is crucial but challenging as natural speech lacks clear boundaries, yet humans perform this computation effortlessly. Speech aligns neural activity to syllabic rhythms, predicting syllable timing, but the underlying cortical mechanisms remain unknown. Relating this macroscopic behavior to neurobiology is challenging; however, next-generation neural mass models promise to resolve this. We demonstrate that these models reproduce sharpness-tuned tracking and acoustic edge extraction. Dynamical analyses indicate this occurs through thresholded phase-resetting to phoneme onsets, triggering cross-frequency nested oscillations. Our results both advance biophysical understanding of syllable segregation and validate the models’ capacity for simulating macroscopic neural activity. These models offer a bridge between the neurobiology of the auditory cortex and speech processing dynamics that phenomenological models cannot provide.

## Introduction

Humans are extremely good at splitting speech sound into syllables. However, it is not a straightforward task because syllables often arrive in a continuous stream without discrete gaps. In this study, we focus on a key mechanism of syllable segregation: the alignment of neural activity with the syllabic rhythm (Räsänen et al., 2018). This “neural speech tracking” has been causally linked to speech comprehension in stimulation studies (Riecke et al., 2018; Zoefel et al., 2018a).

Multiple mechanisms have been proposed for speech tracking and it is unknown whether tracking is the result of activity evoked by acoustic features or phase-resetting of endogenous oscillations. This is an example of a broader debate in sensory neuroscience (Capilla et al., 2011; Zoefel et al., 2018b; Lalor and Nidiffer, 2025). The evoked response hypothesis suggests that neural activity tracks speech by responding to discrete features of the envelope such as syllable onsets. Representations of these features have been identified within the superior temporal gyrus (STG; Hertrich et al., 2012; Oganian and Chang, 2019), and mathematical modeling suggests that evoked responses can account for the response to natural speech seen in macroscale neural recordings (Zou et al., 2021; Oganian et al., 2023).

The phase-resetting hypothesis, in contrast, assumes that pre-existing oscillatory activity within the auditory cortex is shifted by a stimulus, causing entrainment to the stimulus rhythm. Neuronal oscillations have been implicated in various related functions (Schroeder and Lakatos, 2009; Peelle and Davis, 2012; Obleser and Kayser, 2019; Poeppel and Assaneo, 2020), and they may facilitate speech processing through a hierarchical process using cross-frequency coupling to gate neuronal spiking in time with quasi-rhythmic stimuli (Giraud and Poeppel, 2012).

Both of these mechanisms have primarily been investigated using phenomenological models (Doelling et al., 2019; Doelling and Assaneo, 2021; Oganian et al., 2023). Therefore, although we have an idea of the fundamental dynamics responsible for syllable segregation, they are disconnected from the biological structures that support them. To achieve a deeper understanding, we need models that bridge between the high-level processing of syllable segregation and the cortical circuits enacting it. Biophysical models attempt to provide this connection by incorporating known biological details.

To address this, we explore whether modern biophysical neural mass models (NMMs) are a suitable tool for investigating the mechanistic underpinnings of neural speech tracking. Specifically, the Byrne–Coombes next-generation NMM (Byrne et al., 2017; Pietras et al., 2019; Byrne et al., 2020, 2022) is designed to model meso-scale oscillatory circuits. We investigate whether the NMM can reproduce established features of neural speech tracking. To help interpret the NMM’s behavior, we compare its dynamics to phenomenological models of the phase-resetting and evoked response hypotheses.

As the initial benchmark in our study, we use data from Cucu et al. (2022) showing that neural speech tracking is correlated with phoneme sharpness. This result is in agreement with similar findings in the response to non-speech tones (Oganian and Chang, 2019) and spoken digits (Doelling et al., 2014). Further, it has been identified as a primary driver of cortical speech responses (Daube et al., 2019), and shown to normalize to the contextual speech rate (Oganian et al., 2023). This benchmark requires the capture of both gross syllabic rate tracking and the lower-level feature of sharpness-specific tuning.

Here, we recreate the EEG experiment in silico, demonstrating that all three models can reproduce the sharpness-specific tuning of neural speech tracking. While this confirms the NMM’s capacity to track speech, it indicates the benchmark is limited in its ability to distinguish between viable mechanisms. As such, we investigate the models’ dynamics further using the phase concentration metric (PCM; Doelling et al., 2019), a syllabic rate variation test, and inter-event phase coherence (IEPC) analysis. We demonstrate that the NMM tracks the syllabic rate through thresholded phase-resetting and cross-frequency coupling. This is produced by phoneme onsets triggering phase-locked high frequency oscillations, temporally nested within the slower syllabic rate. This behavior qualitatively matches the double peaked IEPC feature previously only recreated with linear evoked models (Oganian et al., 2023) and demonstrates that nonlinear processing of the continuous speech envelope is sufficient to explain the presence of peak-rate event representations in the STG.

## Materials and Methods

### Models

#### Neural mass model

The NMM (Byrne et al., 2017) is an exact mean field reduction of an infinite all-to-all connected network of spiking quadratic integrate and fire neurons. It provides a biophysically grounded description of the activity of a large population of cortical neurons with parameters corresponding to biophysical features such as membrane and synaptic time constants, synaptic reversal potentials, and the distribution of neuron excitabilities. As the model is based on a spiking neural network, it incorporates spike timing and subsequently captures the synchronization of the population rather than just the mean firing rate. We used the first variant (Byrne et al., 2017), incorporating *α*-function synaptic filtering of spikes, but not gap junctions or competing excitatory-inhibitory populations (Pietras et al., 2019; Byrne et al., 2020, 2022). We consider only a single population here, representing a macroscopic ensemble of auditory cortical neurons.

The model comprises a single complex differential equation for a Kuramoto order parameter *Z*(*t*), and two input filters of the synaptic conductance *g*(*t*) and external stimulus current *A*(*t*). The Kuramoto order parameter, *Z*(*t*), describes the synchrony of the population’s spikes and satisfies:

$$C\frac{dZ}{dt}=T_{1}+T_{2}+T_{3},$$

where *C* is the membrane capacitance:

$$T_{1}=-i\frac{(Z(t)-1{)}^{2}}{2},$$

captures the intrinsic, self referential dynamics of the network activity:

$$T_{2}=\frac{(Z(t)+1{)}^{2}}{2}[-\Delta +i[\eta_{0}+A(t)]+iv_{s}g(t)],$$

describes the response to driving forces: the internal excitability *η*_0_ and its heterogeneity Δ, an external drive *A*(*t*), and the global post synaptic current *v*_*s*_*g*(*t*) with *v*_*s*_ the synaptic reversal potential.

The third term, in Equation 1:

$$T_{3}=-\frac{Z{\left(t\right)}^{2}-1}{2}g(t),$$

describes the feedback from state dependent synaptic interaction through the global conductance *g*(*t*).

In Equation 3 above, Δ is a measure of the variation in the natural frequency of the individual neurons which are described collectively by the NMM; specifically Δ is the width of a Cauchy distribution for the natural frequencies. The population excitability is *η*_0_, the center of the Cauchy distribution. *A*(*t*) models the external drive and is a filtered version of the input *s*(*t*), defined by:

$$Q_{D}A(t)=\Pi s(t),$$

where *s*(*t*), the external stimulus, is a linear BSpline interpolation of the envelope, Π is the stimulus amplitude scale and:

$$Q_{D}={\left(1+\frac{1}{\alpha_{D}}\frac{d}{dt}\right)}^{2},$$

where *α*_*D*_ is a timescale for the filtering.

The synaptic input is also defined using a filter:

Qg=kH(Z),

where:

$$Q={\left(1+\frac{1}{\alpha }\frac{d}{dt}\right)}^{2}.$$

Here, *α* is another smoothing timescale, *H*(*Z*) is the measure of neuronal activity:

$$H(Z)=\frac{1}{C\pi }\frac{1-|Z{|}^{2}}{|1+Z{|}^{2}},$$

and *k* is the coupling strength inside the neural mass. For a full derivation, see Byrne et al. (2017).

We used two parameter sets for the NMM, which we refer to as the slow and fast models. For the slow model we increased *C*, and lowered both Δ and *η*_0_ relative to the parameters in the original paper (Byrne et al., 2017). The values were chosen so that the model does not oscillate when there is no stimulation but oscillates at approximately 4 Hz under slight stimulation. The fast model is identical to the model used by Byrne et al. (2017), producing approximately 15 Hz oscillations. Using this original parameter set allows us to investigate what happens if the NMM’s base frequency is distinct from and higher than the stimulus frequency (typically 4 Hz). Table 1 provides the two parameter sets.

**Table 1.** Parameters for the slow and fast NMM models

Parameter	*C* F	Δ mA	*η*_0_ mA	*v*_*s*_ mV	*k*	*α* s^−1^	*α*_*D*_ s^−1^
Slow ≈ 4 Hz	0.066	0.25	1.5	−10	0.105	1/0.035	1/0.0056
Fast ≈ 15 Hz	0.033	0.5	21.5	−10	0.105	1/0.035	1/0.0056

The stimulus amplitude scale Π for the NMM, used to investigate the effect of varying drive strength, was set with reference to the excitability parameter *η*_0_, Π = *η*_0_*D*, where *D* is the drive-excitability-ratio. This allowed us to consider a range of drive strengths that were scaled to the particular internal excitability of the slow and fast models. The mean firing rate generated by the NMM was used to represent its activity. This firing rate is obtained through a conformal transformation of the complex Kuramoto order parameter *Z* to a new order parameter *W* = *πCr* + *iV*, where *r* is the firing rate, *V* is the mean membrane potential (Montbrió et al., 2015), and:

$$W=\frac{1-\bar{Z}}{1+\bar{Z}}.$$

The firing rate is therefore:

r=Re(W)/πC.

Initial conditions for Z∈C were created by sampling its argument arg(*Z*) ∼ Uniform( − *π*, *π*) and magnitude |*Z*| ∼ Uniform(0.0, 0.9) such that it remained in the unit disk centered on the origin. Initial values for *g*, $\dot{g}$, *A*, and $\dot{A}$ were 0.

#### Phase-resetting oscillator model

Our second model is the two-dimensional oscillator used by Oganian et al. (2023) to model phase-resetting. This contrasts with the biophysically derived NMM by representing the algorithmic process of phase-resetting, rather than the biological circuitry that may implement it. It is an oscillator with a fixed natural frequency that reacts to a stimulus only by shifting its phase toward a particular setpoint. The model consists of a Hopf normal-form with external stimulus *s*(*t*) that is modulated by the oscillator’s phase:

$$\begin{array}{rl} \dot{\;\theta } & =2\pi F-\frac{cs(t)}{r}\sin\;\theta ,\\ \dot{r} & =r(1-r^{2})+cs(t)\cos\;\theta , \end{array}$$

with phase *θ*, magnitude *r*, and coupling strength *c*.

This model orbits around the unit circle, with the stimulus forcing a phase-reset toward *θ* = 0. For this model the 10 s stimuli streams were normalized to avoid the effect of varying power in the stimulus. Specifically, for the 60 trials corresponding to a given condition and test, the amplitude was scaled to have a mean per-stream-integral of 20,000 over the samples in the interval [5.0, 10.0] s of the streams. This was simply a value close to the original integral of the first vowel condition trial. In principle, it would be possible to build a multi-oscillator version of this model, however, as for the NMM, a version with only a single macroscale oscillator was tested. We set *c* such that the maximum possible phase correction was equal to *π*; this maximum occurs when *θ* = ±*π*/2 at the maximum value of *s*(*t*), assuming *r* = 1. Thus *c* varies according to the stimulus condition in order to maintain a consistent total “power” of the stimulus across tests.

The activity of the phase-resetting oscillator was taken to be rcosθ following the approach of Oganian et al. (2023). Initial conditions were sampled from random locations on the unit circle, *θ* ∼ Uniform( − *π*, *π*) and *r* = 1.

#### Evoked response model

Our final model was a model of evoked activity. Unlike the NMM or phase-resetting oscillator, this model contains no self sustaining dynamics, instead it acts as a passive filter of an incoming stimulus. The model captures the direct effect of a stimulus on synaptic currents but, unlike the NMM, it does not include any representation of internal circuit dynamics. To ensure consistency between the stimulus processing of the NMM and the evoked model, we use the same linear differential operator *Q*, Equation 8 corresponding to the *α*-function response incorporated in the NMM by Byrne et al. (2017) as a synaptic filter. As such, the response of this model *g*(*t*) to stimulus *s*(*t*) is the solution of:

$${\left(1+\frac{1}{\alpha }\frac{d}{dt}\right)}^{2}g(t)=\Pi s(t).$$

For an impulse-like stimulus, the model responds with a single *α*-function curve with rise time 1/*α*, thus providing a stereotypical evoked response. For a continuous stimulus, this is equivalent to a convolution with an *α*-function kernel. We set *α* such that the rise time was 30 ms. The drive strength Π was set to 1, without loss of generality, as the model is linear with respect to *s*(*t*). Scaling using Π therefore simply rescales the output without affecting its temporal structure. Since we are using the phase-based inter-trial phase coherence (ITPC) for our evaluation, varying Π does not alter the results. Initial conditions were set such that *g* ∼ Uniform(0, 1) and $\dot{g}=0$.

### Sharpness-specific tuning of tracking

**Figure 1.**
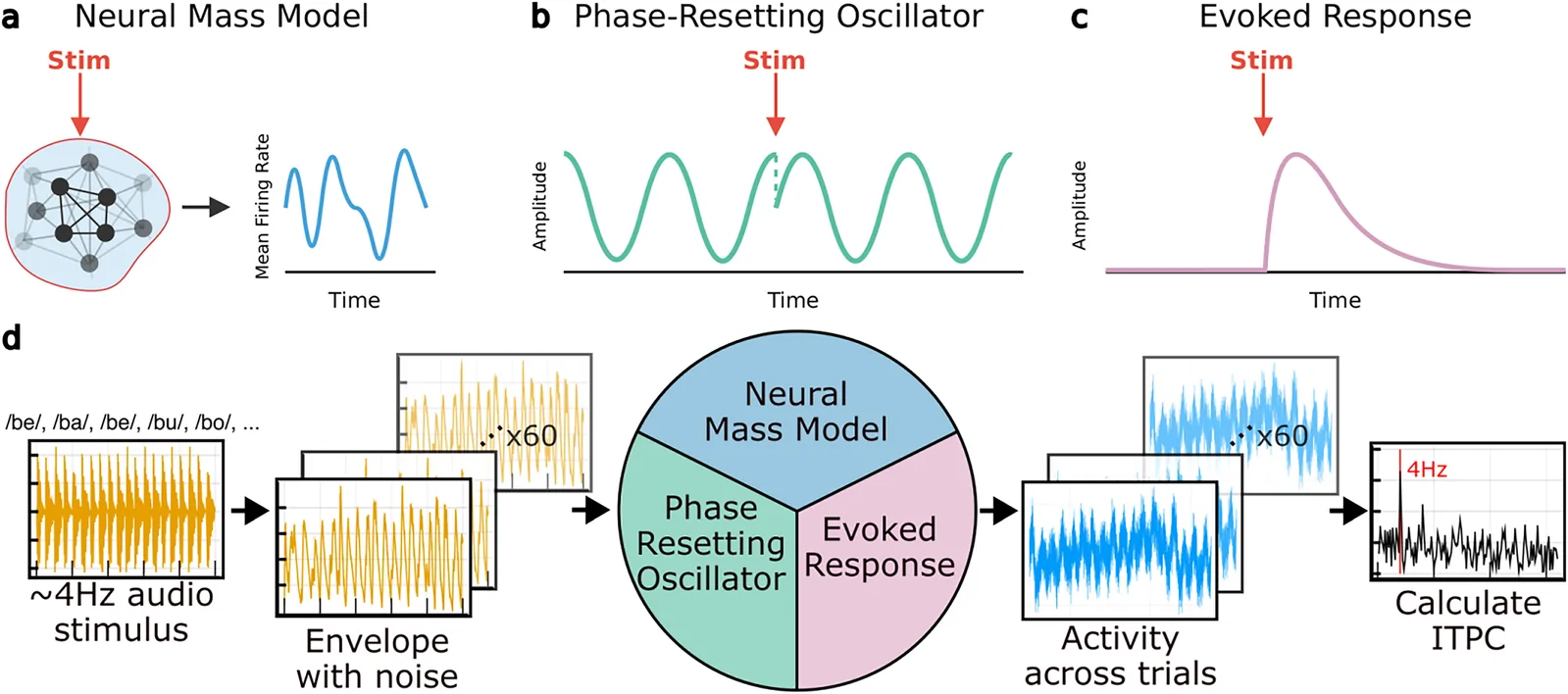
The three models used and the primary simulation design. ***a***, A biophysical neural mass model. This is an exact mean field reduction of a network of spiking neurons, with state corresponding to the mean firing rate of the population. A stimulus is applied to the population as a whole. ***b***, A phase-resetting oscillator. This is a phenomenological benchmark model of one of the hypothesized mechanisms of neural speech tracking. Stimuli act to reset the phase of a state that oscillates at a fixed natural frequency. ***c***, An evoked response model representing the alternative hypothesized mechanism of speech tracking. The model is silent until a stimulus is applied. For an impulse stimulus, a stereotypically shaped response is produced. ***d***, Schematic of our reproduction of Cucu et al. (2022)’s EEG experiment in silico. Raw audio stimuli, consisting of near-isochronous 4 Hz consonant–vowel syllable sequences, are processed into envelopes with noise added to simulate trials. These are used to drive the three models. The inter-trial phase coherence is computed for each model across the trials. The 4 Hz ITPC is then used to investigate the effect of phoneme sharpness on tracking strength. Example model activities are presented in Figure 1-1 in the extended data.

In Cucu et al. (2022), it was demonstrated that the EEG response to syllables is correlated with phoneme sharpness (see, Fig. 6 in their paper). Here we use the same stimuli to investigate whether this experimentally-observed correlation can be recreated in silico (Fig. 1). The following first describes the experimental stimuli, then details how we constructed our in silico stimuli. We then describe the experiments conducted using these stimuli. Finally, we detail parameter sweeps of noise level and drive strength.

10.1523/ENEURO.0379-25.2026.f1-1 Figure 1-1 Example of the response of each model to a given consonant-vowel syllable sequence stimulus. a: Envelope from one of the “b” items with noise, λ = 0.7. b: Response of the slow NMM variant. c: Response of the phase-resetting oscillator. d: Response of the evoked-response model. All responses include post-hoc noise added at a signal to noise ratio of 0.5. Download Figure 1-1, TIF file.

#### Experimental stimuli

The stimuli are near-isochronous 4 Hz auditory streams of consonant–vowel (CV) pairs from 15 different conditions (Cucu, 2021). The 15 conditions are 14 consonant conditions, where the consonants are paired with random vowels in the streams, as well as a vowels only condition. For each condition, there are three distinct auditory streams corresponding to different random vowel sequences that the consonant is paired with, these are hereafter referred to as “items.” For example, one of the “k” items begins

“ke, ka, ki, ku, ke,….”

As such, there are 45 items in total (15 conditions, three items per condition).

#### Stimulus construction

The experimental audio stimuli were transformed to form sound envelopes for each stream. Following the process used in Cucu et al. (2022) to compute the sharpness of the phonemes, we passed the raw auditory data through a cochlear band-pass filter, with frequencies ranging from 80 to 8,000 Hz in 32 log-spaced bands and computed the Hilbert transform to get the analytical signal in each frequency band. These were then summed to give the final envelope. The maximum amplitude of the envelopes was approximately 0.25.

Additive syllable-based noise was included to simulate background “chatter” during the experiment. The noise streams were constructed by squeezing and stretching the existing syllable streams. For each instance of the noise, 20 syllables were selected at random, and these were concatenated and subject to temporal distortion while keeping the overall stimulus length fixed. We produced 60 different instances of this noise, giving 60 trials for each item. To ensure transient effects from the drive were minimized we appended 5 s of syllable-based noise of the same sort to the beginning of each stimulus stream, aligned such that the end of a distorted syllable aligned with the start of the stimulus stream. Overall there were thus 60 trials × 3 items for each of 15 conditions giving, in total, 2,700 distinct 10 s in silico stimuli. We explored the effect of the strength of the noise using a noise-to-stimulus ratio *λ*. For a particular noise-to-stimulus ratio *λ*, the stimulus envelopes were multiplied by (1 − *λ*), before the noise scaled by *λ*, was added to them. This therefore required 2,700 stimuli streams per *λ* setting.

For the evoked model we investigated the response to three variants of the auditory stimuli. These variants were: the original envelopes as used for the NMM and phase-resetting model, the derivative of a 10 Hz low-pass filtered version of the envelopes, and a series of impulses corresponding to peak-rate events. Ten hertz was selected for the low-pass filter as it is the upper end of where the power in the speech modulation spectrum is concentrated (Ding et al., 2017b). We used peak-rate impulses because peak-rate acoustic edge representations have been identified in auditory speech cortex (Oganian and Chang, 2019).

To create the peak-rate impulse stimuli, we smoothed the original envelopes with a 10 Hz low-pass filter and then applied a peak finding algorithm on the derivative of the envelope. This identified the peaks, with a lower threshold of 0.1 peak magnitude. Once the peaks were identified, impulses were created at the time points of the peak events, with heights set to the magnitude of the derivative at that point. The impulses were then scaled to a range of [0.5, 1.0] following the approach of Oganian et al. (2023).

#### Experiments

Using the trial stimuli outlined above, we conducted several in silico experiments to investigate the response of the models and simulate (Cucu et al., 2022)’s experiment.

Specifically, we applied the stimuli to the models as an external drive, and recorded the response. The ITPC was calculated over the 60 trials for a given condition item. For each condition, the mean of its three items ITPCs was then calculated. To compare to the experimental results in Cucu et al. (2022) we then calculate the correlation between this mean ITPC and the phoneme sharpness.

We used latency to maximum derivative, *L*, to measure phoneme sharpness as this was identified to be most strongly correlated with tracking strength by Cucu et al. (2022) amongst a range of acoustic edge features. We used the values derived by Cucu et al., as shown in Table 2. These were obtained by identifying the maximum derivative of the envelope after applying a band-pass filter between 2 and 10 Hz, and then identifying the time taken to reach this point from the onset of the phoneme.

**Table 2.** Latency to peak derivative, *L*, for each stimulus condition

Condition	b	n	m	d	Vowel	g	l	p	t	k	r	v	z	f	s
*L* (ms)	17.01	18.59	19.19	21.63	23.49	29.17	29.98	37.01	47.66	51.07	52.77	64.88	87.62	93.07	98.83

Cells are ordered by increasing *L* and colored according to the phoneme type: vowel, voiced stops, unvoiced stops, nasals, sibilants, liquids, and fricatives.

All models were tested with the envelope stimuli. The evoked model was also tested with the derivative of the envelope, and peak-rate impulse stimuli. We ran these tests for a range of noise-to-stimulus ratios and, for the NMM, we also investigated the effect of the drive strength in relation to the excitability of the model (see the “Simulation settings: noise and drive strength” section below). An example response of each model is presented in the extended data Figure 1-1.

#### Simulation settings: noise and drive strength

To identify reasonable simulated experimental conditions for our tests, in particular the noise level and drive strength, we calculated the concordance correlation coefficient between the model ITPCs and those obtained in Cucu et al. (2022)’s experiment. We wanted to find operating conditions that produced tracking of a similar strength to that observed in Cucu et al. (2022), as well as a similar correlation with phoneme sharpness, taking this to mean we were operating under “realistic” experimental conditions. To be clear, this is not to say we were attempting to fit to biologically plausible values of these parameters, only to approach dynamical regimes that appear qualitatively similar to the response in experiment. The *D* and *λ* parameters effectively represent input signal gain, and background noise levels, and can be thought of as tuning the experimental environment, rather than tuning the behavior of the models themselves.

Specifically, we tested all of the models using noise-to-stimulus ratios *λ* ∈ [0, 0.05, 0.1, 0.3, 0.5, 0.7, 0.9, 1.0]. This ratio was used in the stimulus construction to determine how much noise to add to the stimulus stream. For instance, with *λ* = 0.6 the drive is 60% noise and 40% stimulus. For the NMM we also varied the drive strength. We did this relative to the excitability, *η*_0_, of each of the fast and slow models. To do this, we produced a drive-excitability-ratio parameter *D*, taking log-spaced values between 0.1 and 300, but capped at 100 for numerical stability. As such *D* ∈ [0.1, 0.496, 2.46, 12.2, 60.5, 100.0]. Using this, we then set the drive scale parameter Π = *η*_0_*D*, where *η*_0_ was the excitability of the particular fast or slow model being tested; see the NMM model description, Equation 5, for further detail. For the phase-reset and evoked response models, the drive was not varied. For the phase-reset model we instead used normalization of the stimuli and model parameters to set a consistent scale of response, meaning any changes to the drive scale would get normalized out. For the evoked model the drive strength was fixed to 1, as varying this does not affect the purely phase-based ITPC evaluation. To be clear, the *D* and *λ* parameters effectively represent input signal gain, and background noise levels, and can be thought of as tuning the experimental environment, rather than tuning the behavior of the models themselves.

#### Post hoc noise

In addition to the noise in the stimulus, constructed out of distorted syllables, we added post hoc 1/*f* noise to all of the model activities with a signal to noise ratio of 0.5. This was used to simulate the noise present in EEG. We used the PowerLawNoise.jl (v0.0.1) package to produce the noise (https://github.com/astrogewgaw/PowerLawNoise.jl).

### Model dynamics investigation

Once we had established that the NMM, as well as the phase-resetting oscillator and evoked model, could reproduce the result from Cucu et al. (2022), we ran further experiments to explore the models’ dynamics more closely. First, we used the PCM, which was developed to distinguish between evoked or phase-reset-like responses to rhythmic tones. Then, we re-ran the CV-syllables experiment while varying the syllabic rate of the stimuli. This is a similar test, but reports changes in tracking strength only, rather than the changes in phase lag between stimulus and response that the PCM reports. Finally, we calculated the IEPC of the NMM’s response over a time window centered about the phoneme onsets of a particular condition stream to identify any transient, onset-related dynamics.

#### PCM experiment

The PCM evaluates the similarity of phase lags between the stimulus envelope and model response across stimulus rates. The phase lag is computed from Gaussian filtered versions of the stimulus and response, see “Evaluation metrics and statistical analyses” section for details. The PCM varies between zero and one as it represents the magnitude of the circular mean of unit vectors in the complex plane. For an evoked response model, the PCM is expected to be low, as the phase lag shifts with the stimulus rate. For a phase-resetting oscillator, the PCM is expected to be high as the phase lag remains more consistent under stimulus rate changes.

To use the PCM to investigate the behavior of the NMM, we generated sine wave stimuli of varying frequencies and applied a DC shift of +1 to ensure they were strictly positive. These were used to drive the NMM and phase-resetting oscillator. For the evoked model, unit impulses timed at the peaks of the sine wave were used instead. We varied the stimulus frequency from 2 to 15 Hz in 60 steps and computed the PCM from the resulting trajectories. Details of the PCM implementation are provided in the “Evaluation metrics and statistical analyses” section below. Model parameters were kept the same as for the sharpness-tuning experiment, except the drive amplitude for the NMM, which was fixed at 15.0.

#### Speech rate experiment

To vary the syllabic rate of our stimuli, we scaled the time used to sample from the stimulus interpolation object storing them. In the ODE simulations, the drive was obtained from an interpolation object that returned a value for a given time, i.e., drive=stimulus_interpolator(t). When sampling using the actual time, this provided the 4 Hz stimulus, which was 10 s long. Here, we instead sampled using the real time *t* scaled by a factor *τ*. For instance, for a run with a desired syllabic rate of 1 Hz, *τ* = 0.25, and for a syllabic rate of 12 Hz, *τ* = 3. This necessarily results in different length stimuli, for instance, the 10 s baseline duration would become 5 s when sampling at twice the rate for a 8 Hz syllabic rate. To ensure that the calculation of ITPC was done over a suitable number of cycles, we extended the stimulus period from [5, 10] s to [5, 20] s, simply repeating the compressed or stretched stimulus segment as many times as necessary. This repetition was also done separately for the 5 s of noise that prepended the stimulus to ensure that the noise did not enter the ITPC calculation window. Thus, to avoid transient effects, we set the ITPC calculation window to [5.5, 20] s, skipping the first 0.5 s of the stimulus period.

As we were interested only in how the syllabic rate affected the tracking strength, we kept other parameters constant. For the simulation settings, we chose *D* = 12.2 as this was found to be optimal for the sharpness-tuning experiment and *λ* = 0.3 to ensure strong enough tracking to observe any variation due to syllabic rate. Model parameters were the same as those used in the sharpness-tuning experiments. We ran this test for 20 trials, for one item, of each of the conditions, reporting the mean ITPC across these 15 conditions for each syllable rate.

#### IEPC about phoneme onsets

Following the approach in Oganian et al. (2023), we computed the IEPC over a time window centered at the onset of the phonemes. Specifically, for one of the “b” phoneme condition streams, we identified the peaks in the derivative of a 10 Hz low-pass filtered version of the envelope that occurred between 5 s (the start of the stimulus period) and 9 s, taking these to be the phoneme onsets. This was done for 20 trials, in total, identifying 340 distinct phoneme onsets.

We band-pass filtered the response of the fast NMM to these 20 “b” condition trials with a second order Butterworth filter into 100 frequency bands centered at frequencies uniformly spaced between 0.67 and 35 Hz. Each *f* Hz band was set to *f* ± 1 Hz, except for frequencies below 1 Hz, where the lower bound was set to 0.1 Hz. Zero-phase filtering was used to prevent phase distortion. We applied a Hilbert transform to the band-pass filtered signals to obtain the analytical signal. The instantaneous phase in each frequency band was then taken to be the argument of the analytical signal.

These instantaneous phase values were segmented into time windows centered at each of the 340 phoneme onsets. Each window ranged from −0.25 to +0.25 s about the phoneme onset. Finally, we converted the instantaneous phase values into exponential form, creating complex unit vectors. The magnitude of the mean of these vectors gave the IEPC for each frequency band (see the “Evaluation metrics and statistical analyses” section for formal details). To further identify any transient effects of the phoneme onset, we computed a baseline adjusted IEPC. To do this, we subtracted the mean IEPC value between −0.2 and −0.1 s in each frequency band from the original IEPC values.

Additionally, to explore whether any “ringing” entrainment was present at the syllabic rate after stimulus cessation, we computed the 4 Hz IEPC over all 60 of the “b” condition trajectories from 0 to 20 s, with the first 5 s being noise, 5 to 10 s the “b” syllable condition stimuli, and with the drive amplitude set to 0 from 10 s onwards.

### Evaluation metrics and statistical analyses

#### Inter trial phase coherence

The ITPC of the model activity across the 60 trials for a given stimulus stream was used to measure the alignment to the periodic stimuli, as in Ding et al. (2017a) and Cucu et al. (2022). What is called the ITPC here is often called the mean square resultant, *R*^2^, in more general context. In particular, for a given frequency *f*:

$$R_{f}^{2}={\left|\frac{1}{N}\sum_{n=1}^{N}\frac{c_{nf}}{\left|c_{nf}\right|}\right|}^{2},$$

where *c*_*nf*_ is the *f* Hz Fourier coefficient of the *n*th signal, so that:

$$e^{i\theta_{nf}}=c_{nf}/|c_{nf}|,$$

defines the phase *θ*_*nf*_. Here, *n* is the trial index with *N* trials in total. The mean of the ITPC at 4 Hz across each condition’s test streams was calculated, and used in the computation of the correlation with the phoneme sharpness. The ITPC ranges between 0 and 1, where 0 indicates no coherence or alignment between the *f* Hz frequency components in the trial signals, and an ITPC of 1 indicates perfect coherence or aligned phase.

#### Correlation and multiple comparison corrections

As in the original analysis, Cucu et al. (2022), we measure the correlation between the tracking strength, $R_{f}^{2}$ and the phoneme sharpness. We evaluate the significance of this correlation for each model using a two tailed *t*-test with 13 degrees of freedom (*n* − 2, *n* = 15 conditions). The effect of multiple comparisons is assessed and reported separately for both the Bonferroni correction and the Benjamini-Hochberg FDR correction (with a false discovery rate of 0.05). Confidence intervals are computed for the correlation coefficients using the *z*-transformation. We do this at the simulation conditions that we decided are most realistic through comparison of the mean ITPC across conditions of the models to that of the experimental EEG result (see “Simulation settings” section). However, in the extended data, we report how the ITPCs, correlations, and confidence intervals change as we change the operating parameters for the simulation. Overall, we are seeking to make a qualitative comparison between the response of these models and the experimental EEG result.

#### Concordance correlation coefficient

To identify reasonable experimental conditions for our tests, for instance, noise levels and drive strengths, we compared the model ITPCs across conditions with those identified in experiment using the concordance correlation coefficient *ρ*_*c*_ (Lin, 1989):

$$\rho_{c}=\frac{2\rho \sigma_{x}\sigma_{y}}{\sigma_{x}^{2}+\sigma_{y}^{2}+(\mu_{x}-\mu_{y}{)}^{2}},$$

where *ρ* is the correlation coefficient, $\sigma_{x}^{2}$ and $\sigma_{y}^{2}$ are the variances of the datasets, and *μ*_*x*_ and *μ*_*y*_ are the means of the datasets. *ρ*_*c*_ assesses the agreement between datasets, accounting for bias and scale as well as correlation by quantifying how far the data deviate from each other, or equivalently the line *y* = *x*, on a plot of *x* versus *y*.

#### Phase concentration metric

To compute the phase lag between the stimulus and response at a particular stimulus rate *f*_*s*_, the time series were first filtered using a Gaussian kernel in the frequency domain with center *μ* = *f*_*s*_ and width *σ* = *f*_*s*_/2.

If *X*(*f*) is the Fourier transform of the signal [the response, *r*(*t*) or stimulus envelope, *s*(*t*)], then the filter kernel:

$$H(f)=\exp\;\left(-\frac{1}{2}{\left(\frac{|f|-\mu }{\sigma }\right)}^{2}\right),$$

and the filtered signal *x*(*t*) is obtained via the inverse Fourier transform of the pointwise product:

$$x(t)=F^{-1}\{X(f)\cdot H(f)\}.$$

The instantaneous phases, *ϕ*_*r*_(*t*) and *ϕ*_*s*_(*t*), are then calculated by applying a Hilbert transform to the filtered signals. The phase lag time series then follows as:

$$\Delta \phi (t)=\phi_{r}(t)-\phi_{s}(t).$$

Finally, these phase lags are transformed into complex unit vectors and their mean taken to produce a mean phase lag vector for the stimulus rate:

$$V(f_{s})=\frac{1}{T}\sum_{t=1}^{T}\exp\;(i\Delta \phi (t)).$$

The PCM is then the magnitude of the mean of these vectors across stimulus rates:

$$\mathrm{PCM}=\left|\frac{1}{N_{f}}\sum_{1}^{N_{f}}V(f_{s})\right|.$$

#### Inter event phase coherence

The fundamental calculation of the IEPC is similar to that used for the ITPC, however there are several key differences. For the ITPC, we calculate the circular mean of complex unit vectors defined by the *f* Hz Fourier coefficients across *N* trials for a given stimulus stream. For the IEPC, we instead calculate the circular mean of complex unit vectors obtained from the instantaneous phase of Hilbert transformed band-pass filtered signals, across the *N* = 340 time windows centered at the phoneme onsets. Thus for a set of instantaneous phases *ϕ*_*nf*_(*t*) from the analytic signal in frequency band *f* over windows *n* ∈ [1, *N*] we have:

$$\text{IEPC}(f,t)=\left|\frac{1}{N}\sum_{n=1}^{N}e^{i\phi_{nf}(t)}\right|.$$

### Code accessibility

The code/software and data described in the paper is freely available online at https://github.com/Modelling-Speech-Processing/Shannon_et_al, and the code is also provided in Extended Data 1. In addition, the code and data are available at the University of Bristol data repository, data.bris, at https://doi.org/10.5523/bris.2796nqydg7fub27scai9muahlr.

10.1523/ENEURO.0379-25.2026.d1 Data 1 Custom Julia scripts used to generate the stimuli, define each of the models, run each experiment, compute relevant statistics, and plot all of the figures. Download Data 1, ZIP file.

## Results

**Figure 2.**
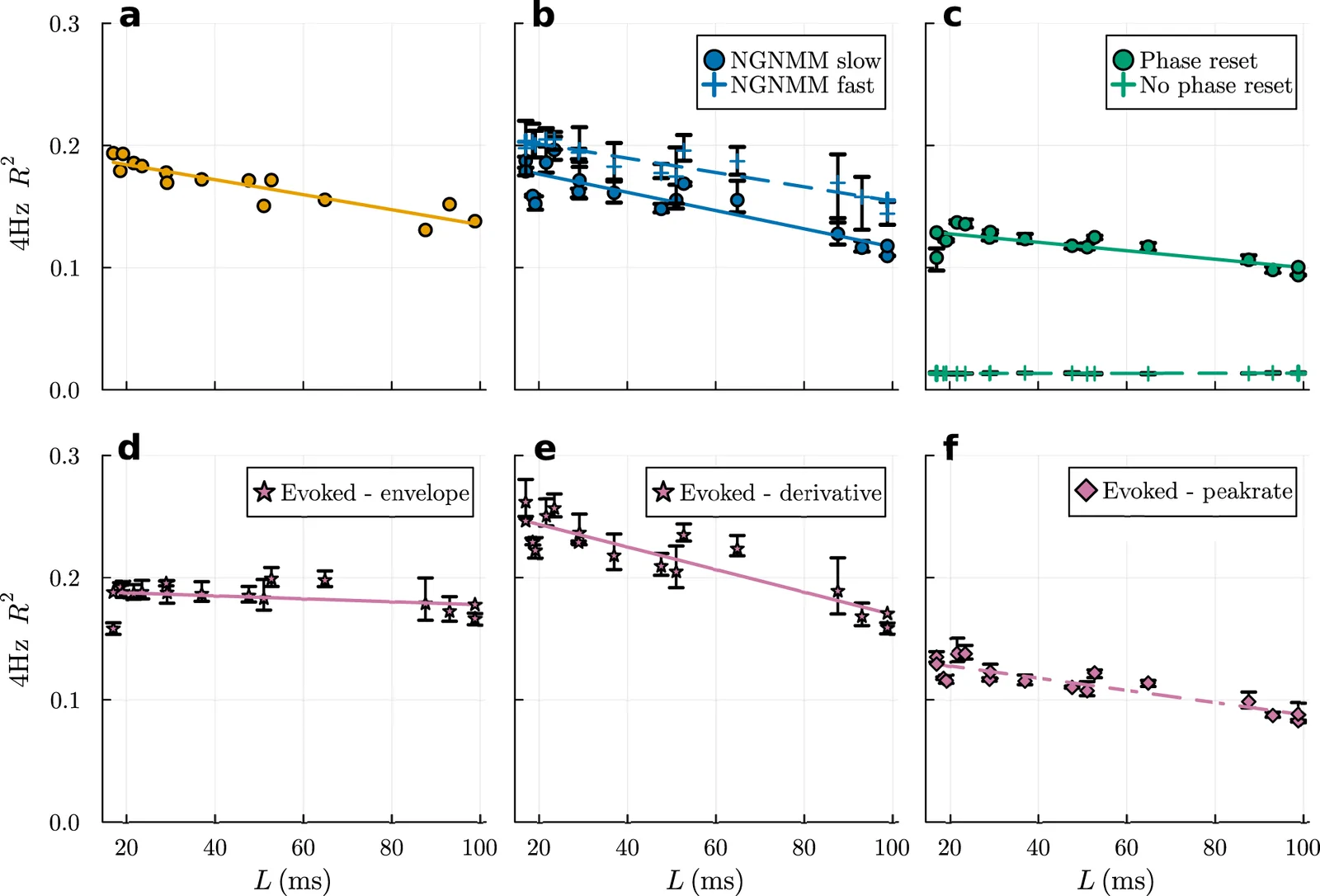
Mean 4 Hz ITPC across test streams versus envelope sharpness as measured by the latency to maximum derivative *L*. Each point represents a different consonant–vowel condition. ***a***, Experimental EEG result from Cucu et al. (2022). ***b***, 4 Hz coherence of the NMM response under the two parameter settings, fast (≈15 Hz) and slow (≈4 Hz). ***c***, 4 Hz coherence of the phase-resetting oscillator response, with and without phase-resetting. ***d***, 4 Hz coherence of the evoked response to the stimulus envelope. ***e***, 4 Hz coherence of the evoked response to the derivative of the envelope. ***f***, 4 Hz coherence of the evoked response to the peak-rate event impulse train stimuli. Error bars show the range of the 4 Hz ITPC over the three test items for each condition.

At representative operating conditions (*λ* = 0.9, *D* = 12.2), both the NMM and the phase-resetting oscillator recapitulate the correlation from Cucu et al. (2022), [*r*(13) = −0.91, *p* < 0.001], Figure 2*a*–*c*. This is the case for both the slow [*r*(13) = −0.85, *p* < 0.001, 95% CI [ − 0.95, − 0.61]] and fast [*r*(13) = −0.91, *p* < 0.001, 95% CI [ − 0.97, − 0.74]] parameter combinations of the NMM. Overall, the ITPC is greater for the slow version of the model. This may be due to the alignment of the baseline oscillations with the frequency of the 4 Hz stimulus. The phase-resetting oscillator model also successfully reproduces the negative correlation when driven by the envelope: *r*(13) = −0.79, *p* = 0.0005, 95% CI [ − 0.93, − 0.46].

The evoked model does not respond in the same way to the stimulus envelope. Unlike for the NMM and phase-reset models, it does not create a negative correlation between sharpness and tracking strength in the response of the evoked model, *r*(13) = −0.30, *p* = 0.270, 95% CI [ − 0.71, 0.25], Figure 2*d*. This is despite still causing the 4 Hz activity to align across trials, with a mean 4 Hz ITPC = 0.184.

However, the evoked model does produce the negative correlation observed experimentally when driven by the pre-processed forms of the stimulus. Specifically, with the derivative of the stimulus envelope, *r*(13) = −0.88, *p* < 0.0001, 95% CI [ − 0.96, − 0.67] and with the peak-rate impulse stimulus, *r*(13) = −0.87, *p* < 0.0001, 95% CI [ − 0.96, − 0.64], Figure 2*e*, *f*. The evoked model demonstrates the negative correlation for all but the envelope-based stimulus. Overall, under this set of simulation conditions, *λ* = 0.9, and, for the NMM, *D* = 12.2, the magnitude of the 4 Hz ITPCs closely resemble the experimental result.

**Figure 3.**
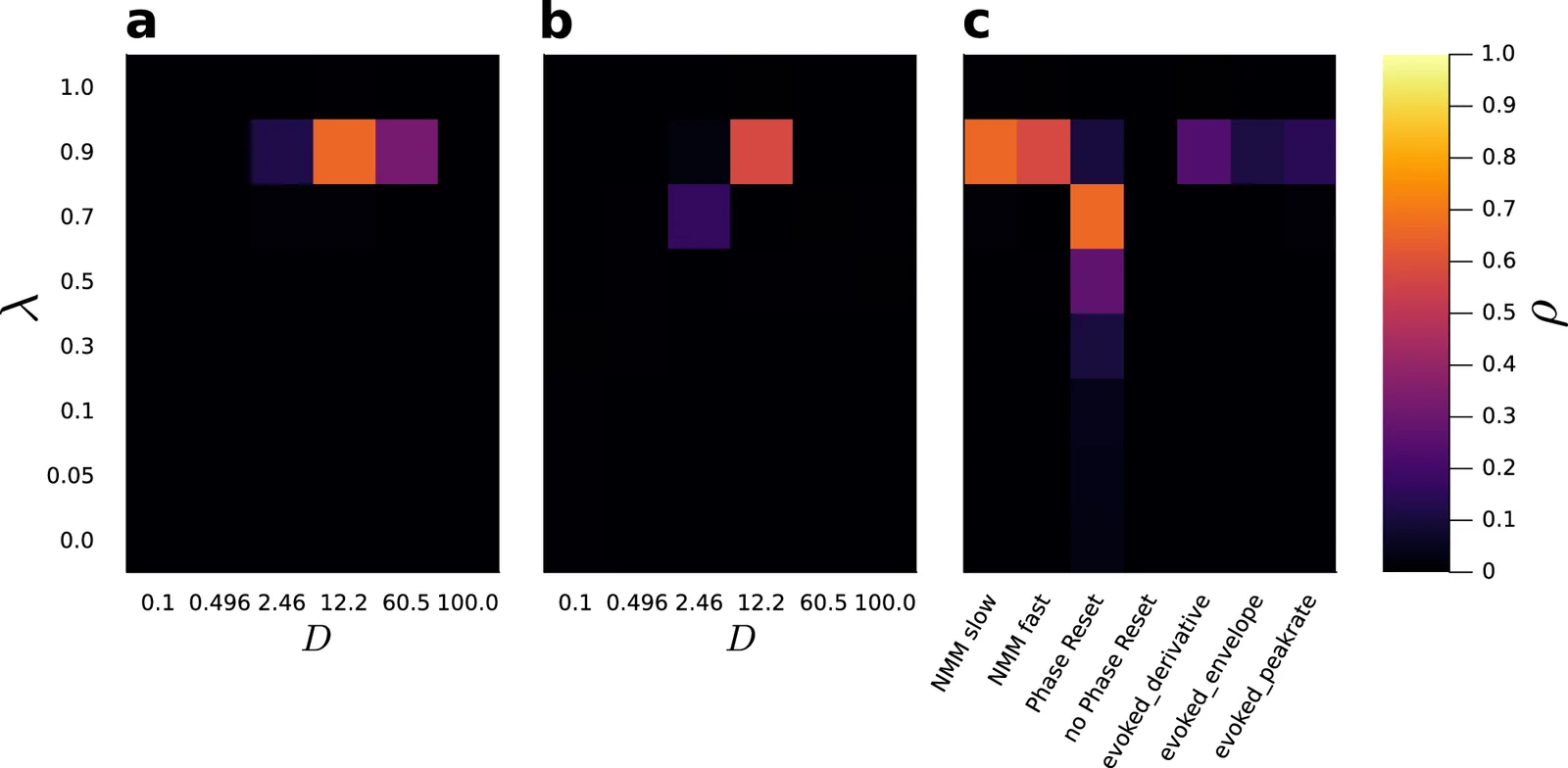
Heatmaps of the concordance correlation coefficient, *ρ*, between the mean ITPC, *R*^2^, per phoneme condition of the models and the EEG data. ***a***,***b***, The slow and fast NMM respectively, as *D* and *λ* are varied. ***c***, For each model as *λ* is varied. The admissible range of *ρ* values is between 0.0 and 1.0, with 1.0 indicating a perfect match between the models *R*^2^ values and those obtained from the EEG (Cucu et al., 2022). The full, per-phoneme-condition, ITPCs for each model under every experimental condition are presented in Figures 3-1 and 3-2, alongside the corresponding correlations and *p* values in Figures 3-3 to 3-6.

Note that the high amount of noise in the stimulus was required to obtain this similarity. This is made evident by observing the concordance correlation coefficient between the EEG based ITPCs and each model’s ITPCs as the simulated noise strength *λ* is increased (Fig. 3*c*).

10.1523/ENEURO.0379-25.2026.f3-1 Figure 3-1 Mean 4 Hz R2 across each phoneme condition’s three items vs L for both the slow (blue) and fast (orange) NMM models. Each subplot presents the results across values of D, with the colour intensity increasing with increasing D, for a particular λ. EEG result from Cucu et al. (2022) included for comparison. Higher D generally increases the entrainment strength. A sufficient level of noise λ is required to obtain an entrainment strength comparable to EEG experiment. Download Figure 3-1, TIF file.

10.1523/ENEURO.0379-25.2026.f3-2 Figure 3-2 Mean 4 Hz R2 across each phoneme condition’s three items vs L for all of the models tested in this work. Each subplot presents the results for a particular λ. NMM results are for D = 12.2. EEG result from Cucu et al. (2022) included for comparison. Download Figure 3-2, TIF file.

10.1523/ENEURO.0379-25.2026.f3-3 Figure 3-3 Correlation between phoneme sharpness and 4 Hz ITPC for the fast NMM variant across λ and D values. We present uncorrected and corrected p values for the correlations. Bonferroni and Benjamini-Hochberg FDR corrections were calculated for eight comparisons (the model and stimulus variants) and a false discovery rate of 0.05. Download Figure 3-3, DOCX file.

**Figure 4.**
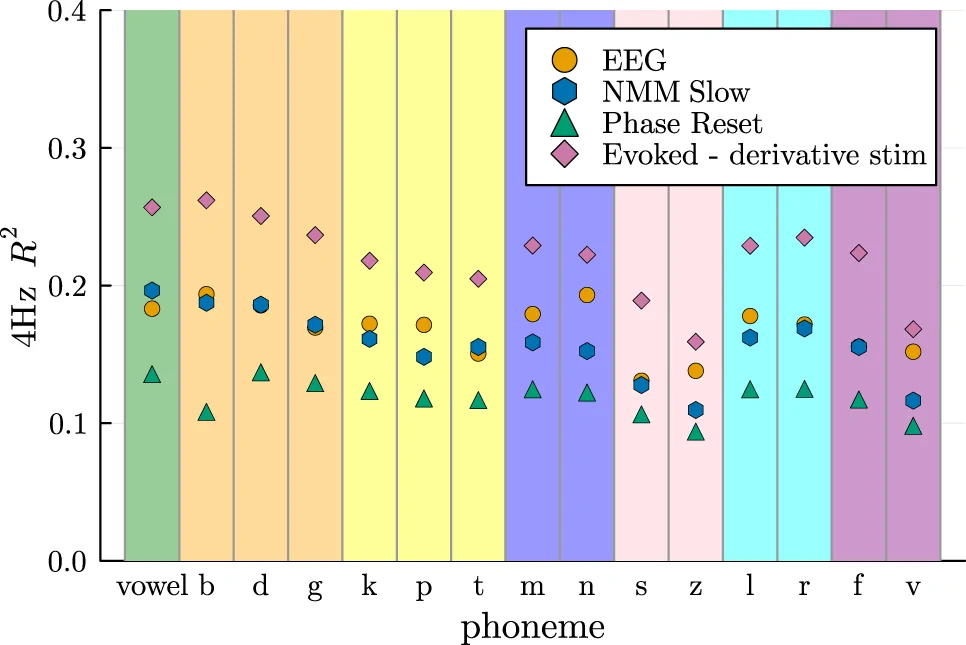
Mean 4 Hz ITPC versus phoneme condition for each model alongside the experimental EEG result. Each point represents a different consonant–vowel condition. Vertical bands are colored according to the phoneme type: vowel, voiced stops, unvoiced stops, nasals, sibilants, liquids, and fricatives.

10.1523/ENEURO.0379-25.2026.f3-4 Figure 3-4 Correlation between phoneme sharpness and 4 Hz ITPC for the slow NMM variant across λ and D values. We present uncorrected and corrected p values for the correlations. Bonferroni and Benjamini-Hochberg FDR corrections were calculated for eight comparisons (the model and stimulus variants) and a false discovery rate of 0.05. Download Figure 3-4, DOCX file.

**Figure 5.**
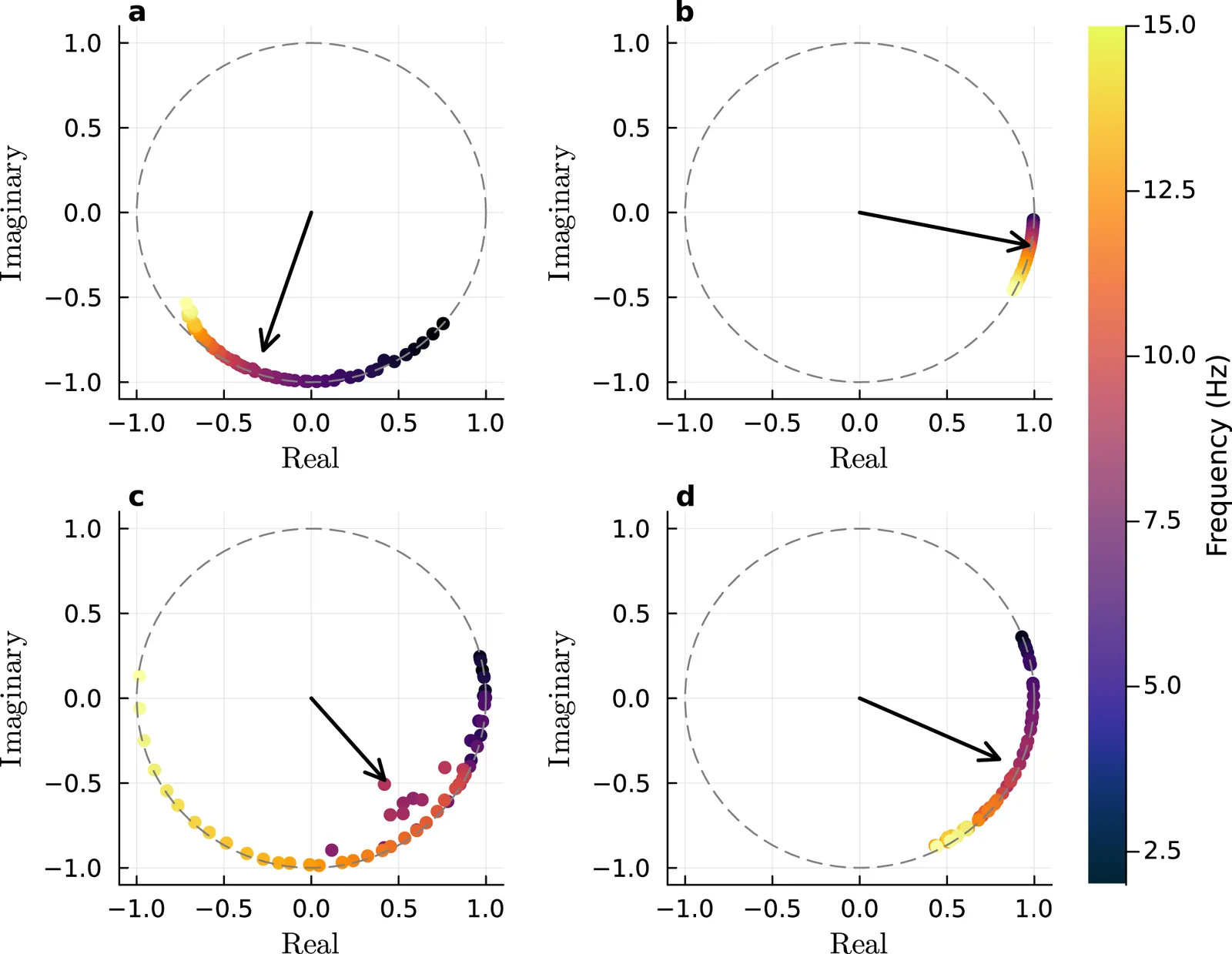
Phase concentration vectors for each model in the PCM test. ***a***, The evoked model. ***b***, A tuned phase-resetting oscillator. ***c***, The slow NMM. ***d***, The fast NMM. Each vector, represented by a dot, is the mean phase lag across a single response to the stimulus, at a frequency defined by the color. The phase-resetting oscillator, and NMM’s were driven by sine waves. The evoked model was driven by unit impulses aligned to the peaks of the same sine waves. Evoked responses result in a shifting phase lag as stimulus frequency changes. Phase-resetting results in a more consistent phase lag. The PCM, the magnitude of the circular mean of the phase concentration vectors, is indicated by the black arrows.

10.1523/ENEURO.0379-25.2026.f3-5 Figure 3-5 Correlation between phoneme sharpness and 4 Hz ITPC for the evoked-response model for each stimulus variant across λ values. We present uncorrected and corrected p values for the correlations. Bonferroni and Benjamini-Hochberg FDR corrections were calculated for eight comparisons (the model and stimulus variants) and a false discovery rate of 0.05. Download Figure 3-5, DOCX file.

**Figure 6.**
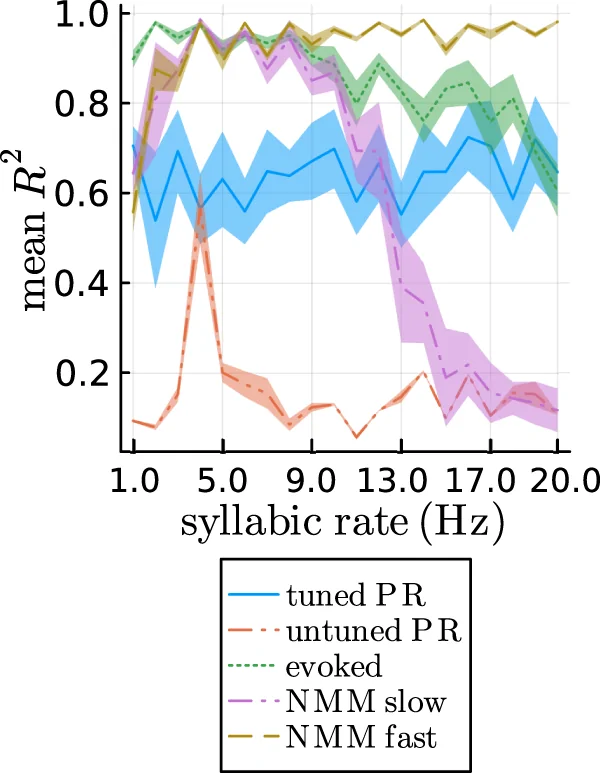
Mean ITPC, *R*^2^, across all phoneme condition items, as the syllabic rate is varied. Blue line, a phase-resetting oscillator tuned so that its own natural frequency matches the syllabic rate, Red line, a phase-resetting oscillator with 4 Hz natural frequency. Green line, the evoked model. Purple line, the slow NMM. Gold line, the fast NMM. Shaded regions represent one standard deviation.

10.1523/ENEURO.0379-25.2026.f3-6 Figure 3-6 Correlation between phoneme sharpness and 4 Hz ITPC for both the phase-resetting and non phase-resetting oscillators across λ values. We present uncorrected and corrected p values for the correlations. Bonferroni and Benjamini-Hochberg FDR corrections were calculated for eight comparisons (the model and stimulus variants) and a false discovery rate of 0.05. Download Figure 3-6, DOCX file.

For the NMM and evoked model, *ρ* is highest when *λ* = 0.9, reaching 0.66, 0.57, and 0.24 for the slow NMM, fast NMM, and derivative driven evoked model respectively. For the phase-resetting oscillator *ρ* is highest at *λ* = 0.7, reaching 0.67. Of course, for *λ* = 1.0, 100% noise, the tracking strength becomes negligible, and any correlation with phoneme sharpness is obscured. For lower values of *λ* we found that the ITPCs became too high. At a point that varied between models (Fig. 3-2), the 4 Hz ITPCs saturated close to 1.0, indicating complete coherence, and again obscuring any correlation with phoneme sharpness. This is the cause of the very low values of *ρ* for lower noise levels. The phase-resetting oscillator required slightly more signal strength to entrain, resulting in the peak *ρ* at *λ* = 0.7 and the lower 4 Hz ITPC’s in Figure 2 at *λ* = 0.9 in comparison to the experiment. The correlation is still present however. The other experimental condition we needed to set was the drive strength for the NMM. Figure 3*a*, *b* present the concordance correlation coefficient between the EEG based ITPCs and the slow and fast NMM’s ITPCs respectively as both the drive strength, *D*, and noise level, *λ*, were varied. It is clear that *D* = 12.2 was the optimum drive-excitability ratio for accurate reproduction of Cucu et al.’s correlation. The full set of ITPCs for each phoneme condition for the slow and fast NMM models across *D* settings are presented in the extended data Figure 3-1.

All of the models except the non-phase-resetting oscillator, and the envelope-driven evoked model produce significant uncorrected correlations (*p* < 0.001, Table 3). This remains the case when corrected using a Benjamini-Hochberg FDR correction with a false discovery rate of 0.05. Under the more stringent Bonferroni correction, the phase-resetting oscillator correlation no longer reaches *p* < 0.001. A full set of correlations and related statistics for all models across all *λ* and *D* settings are provided in the extended data Figures 3-3 to 3-6.

**Table 3.** Correlation between phoneme sharpness and 4 Hz ITPC for each model variant and the original data obtained by Cucu et al. (2022)

Model	Version	*r*	95% CI	*p*	*p*_Bonferroni_	*p*_FDR_
EEG	–	−0.913	−0.97, −0.75	2.0 × 10^−6^	–	–
NMM	Fast	−0.909	−0.97, −0.74	2.7 × 10^−6^	2.2 × 10^−5^	2.2 × 10^−5^
	Slow	−0.854	−0.95, −0.61	5.2 × 10^−5^	4.1 × 10^−4^	1.0 × 10^−4^
Oscillator	Reset	−0.786	−0.93, −0.46	5.2 × 10^−4^	0.0042	6.9 × 10^−4^
	No reset	0.165	−0.38, 0.62	0.56	1.0	0.56
Evoked	Envelope	−0.303	−0.71, 0.25	0.27	1.0	0.31
	Derivative	−0.879	−0.96, −0.67	1.6 × 10^−5^	1.3 × 10^−4^	6.4 × 10^−5^
	Peak-rate	−0.870	−0.96, −0.64	2.5 × 10^−5^	2.0 × 10^−4^	6.8 × 10^−5^

We present uncorrected and corrected *p* values for the correlations. Bonferroni and Benjamini-Hochberg FDR corrections were calculated for eight comparisons (the model and stimulus variants) and a false discovery rate of 0.05.

It is clear that the NMM produces ITPCs that most closely resemble the experimental ITPCs (Fig. 4) for this set of simulation conditions (*λ* = 0.9, *D* = 12.2). However, although overall the model shows a roughly correct negative correlation between sharpness and tracking strength, on a per-phoneme basis there is still some disagreement on the exact coherence, as well as a difference in the exact ordering of phonemes. For instance, the vowel condition has the third highest ITPC for the EEG experimental result, but creates the highest ITPC for the slow NMM. The phase-resetting model would more closely match the EEG result with *λ* = 0.7 (Fig. 3), however we are comparing the models here under the same noise condition. The main point of disagreement between the experimental and simulated NMM results is in the ITPC of the “p,” “m,” “n,” “z,” and “v” conditions. The successful recreation of the negative correlation between sharpness and tracking strength seems to be driven by the ITPC of the models being accurate, in their coherence relative to the other conditions, for the vowel and sibilant conditions that lie at either end of the sharpness scale.

Although all three models seem to be able to reproduce the correlation from Cucu et al. (2022), their mechanisms differ. The two phenomenological models have known mechanisms that are inherent to their construction, these being evoked responses and phase-resetting. However to understand the NMM’s behavior further, we carried out investigations into what kind of mechanisms support its tracking of the CV-syllable stimuli.

The first of these tests was the PCM test, assessing how the phase lag between the stimulus and the model response’s stimulus rate frequency component changes with the stimulus rate. The PCM was constructed such that an evoked model would exhibit a mean phase lag that changes as the stimulus frequency changes, and such that oscillating models that phase-reset have a more consistent, concentrated phase lag as the stimulus frequency changes (Doelling et al., 2019). Our evoked model and phase-resetting oscillator demonstrate this (Fig. 5*a*,*b* respectively). Using these as a baseline example of possible behavior, we can see that both the slow and fast NMM’s look most similar to the evoked model. In particular, although all the models display a systematic relationship between phase lag and frequency, this has higher variance for the NMM and evoked models.

We also examined how the models respond to different syllable rates by using the CV-syllable-based drive with a varying rate. Here we report the mean *R*^2^ at the stimulus rate across 60 trials across all conditions, similar to the sharpness-specific tuning experiment. However we have manipulated the syllabic rate to values between 1 and 20 Hz (Fig. 6). The “untuned” phase resetting oscillator, with a fixed natural frequency of 4 Hz shows a clear peak in coherence at 4 Hz as expected. When this model is set such that it oscillates at the syllabic rate, it successfully entrains across all rates, showing high *R*^2^ across all rates. In contrast, the evoked response model entrains strongly at low frequencies and maintains this across the tested frequencies with a slight fall as the rate increases. At higher frequencies, the low ITPC is possibly due to the timescale of the evoked response being too large for these frequencies. Interestingly and in contrast to the PCM experiment, the slow and fast NMM’s no longer resemble the evoked model. Instead, the slow NMM has a peak centered at its natural frequency like the untuned phase-resetting oscillator, but much broader, and the fast NMM demonstrates high ITPC across all syllabic rates. This is most similar to the tuned phase-resetting oscillator, despite the NMM not having its natural frequency tuned to match the syllabic rate.

**Figure 7.**
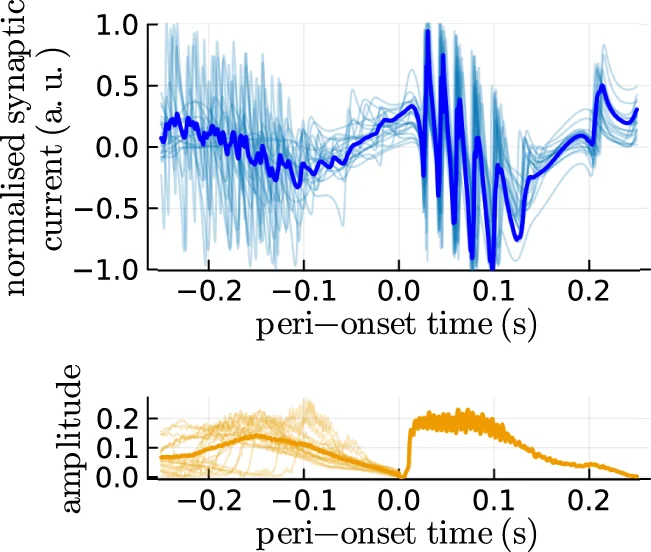
Response of the fast NMM to the initial phoneme onset over 20 trials of the “b” phoneme condition. Despite having random phases at the onset of the stimulus, the trajectories align due to phase-resetting. Noise-to-stimulus ratio *λ* = 0.0 for clarity.

**Figure 8.**
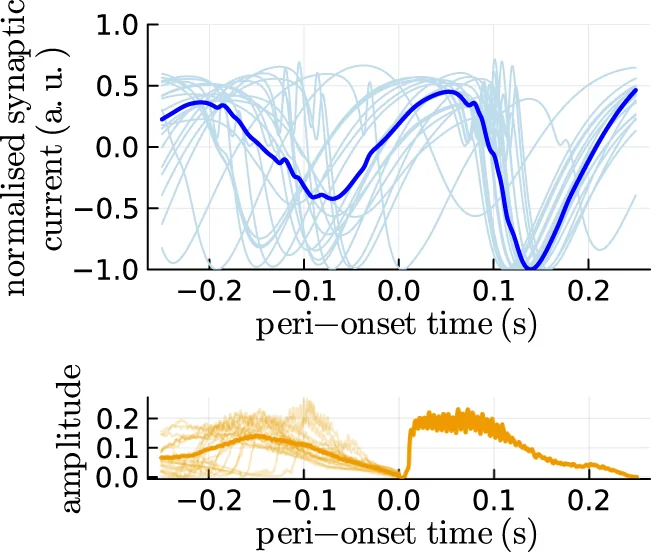
Response of the slow NMM to the initial phoneme onset over 20 trials of the “b” phoneme condition. Despite having random phases at the onset of the stimulus, the trajectories align due to phase-resetting. Noise-to-stimulus ratio *λ* = 0.0 for clarity.

The PCM and syllabic rate tests provide conflicting conclusions. The PCM test suggests that the slow and fast NMMs produce evoked responses. The syllabic rate test suggests that the slow NMM approximates an untuned phase-resetting oscillator while the fast NMM is most similar to the tuned phase-resetting oscillator. To further characterize their behavior, we inspected the NMMs’ responses to individual CV-syllables. Figure 7 presents the response of the fast NMM to the first syllable in the stimulus period, after 5 s of syllable-based noise are applied. Twenty trial trajectories are plotted with the mean trajectory overlaid. It is clear that before the phoneme onset at time 0.0 s, the trajectories are desynchronized due to the noise. After the phoneme onset their phase is reset and they become temporally aligned. This indicates that the fast NMM is being phase-reset by the onset of the phoneme envelopes. It seems that the fast oscillations are being gated by or nested within the 4 Hz syllabic rate oscillations. The phoneme onsets also cause phase-resetting in the slow NMM (Fig. 8). However, no nested oscillations or cross-frequency coupling occurs as the syllabic rate matches the natural frequency of this model. We use the terms cross-frequency coupling and gating here in the nonlinear dynamical systems sense rather than to describe interactions between separate neural populations. Specifically, this refers to nonlinear resonances between frequencies within a single state of a nonlinear system: the slow 4 Hz drive with its sharp phoneme onsets elicits bursts of high frequency activity. These are temporally gated by the envelope of the syllables as the decrease in drive along the tail of the envelope eventually suppresses the fast oscillations.

**Figure 9.**
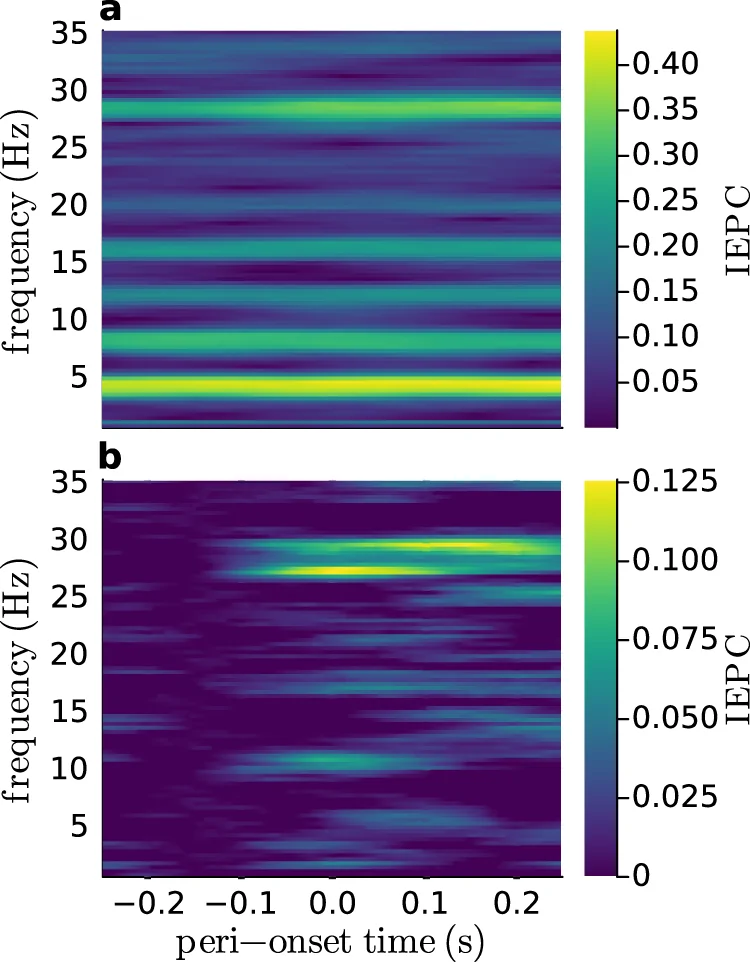
IEPC spectrograms showing transient phase-locking response to phoneme onsets. ***a***, IEPC over all phoneme onsets of the “b” condition. ***b***, IEPC minus the mean IEPC in the time range −0.2 to −0.1. There is strong 4 Hz (and its harmonics) coherence across all phonemes due to the periodic nature of the stimulus. In addition to this, there is high phase coherence at around 30 Hz caused by the phase-reset fast oscillations. The baseline-removed plot illustrates that this is transient and locked to the onset of the phoneme. An exploration of the 4 Hz IEPC after phoneme offset is also presented in the extended data Figure 9-1.

To further probe the fast NMM’s gated oscillations, we computed the IEPC across a 0.5 s window centered at the onset of each phoneme in the stimulus period (5.0–10.0 s) of the 20 trials of the first “b” condition item. Figure 9*a* presents the IEPC across these 340 phoneme onsets and *b* presents the IEPC with the mean IEPC between −0.25 and −0.15 s of each frequency band subtracted to show the transient response. There is a high IEPC at 4 Hz and its harmonics due to the entrainment to the stimulus rate, alongside a high frequency peak at around 28.5 Hz. This high frequency peak is revealed to be a transient response to the phoneme onset in Figure 9*b*, where the IEPC, with a baseline from before the onset subtracted, contains only high IEPC at high frequency post stimulus onset.

10.1523/ENEURO.0379-25.2026.f9-1 Figure 9-1 4 Hz IEPC across the 60 ‘b’ condition trials. The first 5.0 seconds are the noisy syllable based drive, which changes to the 4 Hz drive with λ = 0 from 5.0 to 10.0 s. From 10.0 seconds onwards, the drive strength is set to zero. Blue line, the raw 4 Hz IEPC. Black dotted line, the isochronous stimulus onset point. Red dashed line, the stimulus offset point. Purple thicker line, a 1.2 s window zero-phase moving average smoothed version of the 4 Hz IEPC. Once the isochronous stimulus starts, the NMM is entrained to the stimulus. At cessation, the entrainment drops back to the noisy baseline level indicating no ringing in the entrainment at the syllabic rate post stimulus offset. Download Figure 9-1, TIF file.

Finally, we conducted an exploratory analysis of the 4 Hz IEPC for the “b” syllable condition with the stimulus drive strength set to zero after 10 s. This was to identify if the entrainment at the syllabic rate was sustained or transient post stimulus offset. We found that the 4 Hz IEPC rapidly decreases after the end of the stimulus period (see extended data Fig. 9-1). This indicates that the NMM only transiently entrains at the syllabic rate, similar to the experimental MEG findings of Oganian et al. (2023).

## Discussion

Our results show that the experimentally-observed sharpness-specific tuning of neural entrainment to rhythmic speech can be reproduced using all three models considered here. The first model was a biophysically-inspired neural mass model, the NMM. The other two models were phenomenological, representing the phase-reset and evoked response mechanisms that have been proposed as sources of neural speech tracking. Although both NMMs and both phenomenological models reproduced the correlation, they did so with differing stimulus requirements. The evoked model needed a pre-processed form of the stimulus, for instance, the peak-rate impulses. The NMM and phase-resetting oscillator, however, succeeded when processing just the continuous envelope of the syllables. While these models therefore appear to provide a more parsimonious explanation of sharpness-specific tuning of neural speech tracking, this contradicts the neurophysiological observation that auditory cortex predominantly represents acoustic edges (peak-rate events), rather than the continuous envelope (Oganian and Chang, 2019). As such, this result alone is not enough to decide between evoked or phase-resetting mechanisms, nor to conclude that the NMM and phase-resetting oscillator are better models of sharpness-specific tuning.

To resolve this ambiguity, we moved beyond just matching high-level correlations in the response of the models and explored their dynamics. In the spirit of Doelling and Assaneo (2021), we aim to identify specific biophysical mechanisms that may underpin these processes. Our tests probing the dynamics of the models provide insight into a set of behaviors that suggest that the behavior of the NMM and phase-resetting oscillator are distinct and that the NMM is an excellent candidate for biophysical modeling of neural speech processing.

First, we found in the PCM test that both the slow and fast NMMs had phase lags that varied as the stimulus frequency varied. This was in contrast to the concentrated phase lags produced by the tuned phase-resetting oscillator. A shifting phase lag in the PCM test is indicative of evoked-like behavior, which is somewhat surprising for the NMM given its oscillatory nature. While the tuned phase-resetting oscillator entrains to the gradual modulations of the sine wave, the NMM appears to exhibit a thresholding effect for phase-resetting that causes the evoked-like behavior in this test. When driven by the slowly varying sine waves, the NMM does not phase-reset, and is instead smoothly-modulated, resulting in the shifting phase lag. This suggests that the NMM can act like a linear filter for smoothly-varying signals. This is made evident in the syllabic rate test and subsequent investigation into the phase-resetting dynamics of the NMM.

The evidence for the thresholded phase-resetting dynamics comes from the different result in the syllabic rate test. Here the NMMs behave most like a phase-resetting oscillator. In particular, the slow NMM exhibits a peak in ITPC centered at its own natural frequency. This is similar to the sharp 4 Hz ITPC peak of the 4 Hz phase-resetting oscillator, but broader, indicating that the slow NMM can entrain to syllabic rates that diverge from its natural frequency. The fast NMM exhibits high ITPC across a broad range of frequencies that matches or outperforms the tuned phase-resetting oscillator within the tested frequency range. In this test, the models are driven by the CV-syllable envelopes that were used for the sharpness-specific tuning experiment. These contain broadband energy and sharp onsets that are sufficient to cause phase-resetting in the NMMs where the smooth narrowband sine wave stimuli do not. Figures 7 and 8 demonstrate phase-resetting in both the slow and fast NMMs.

The fast NMM is able to track widely varying syllabic rates, similar to evoked models and the tuned phase-resetting oscillator. A possible explanation for this is the cross-frequency coupling between the slow syllabic rate and the fast intrinsic rhythm that is visible in Figure 7. This may allow even slow syllabic rates to strongly entrain the fast NMM as the sharp onsets of the phonemes trigger phase-reset high frequency oscillations that are gated by the slow syllabic rate. Faster syllabic rates presumably may just entrain the baseline oscillation of the model, resulting in the broad peak of ITPC for the fast NMM. Recent views have stressed the importance of using non-isochronous stimuli when evaluating the algorithms of neural speech tracking to not unjustifiably favor oscillatory mechanisms that rely upon resonance at a fixed frequency (Lalor and Nidiffer, 2025). Although our syllabic rate test used effectively isochronous stimuli, the fast NMM’s robust entrainment across rates suggests that it may also be able to entrain to natural speech’s variable rate. This is in contrast to the simple phase-resetting oscillator, where the rapid loss of entrainment at rates distinct from 4 Hz demonstrates its dependence upon the rhythmic nature of our stimuli.

This cross-frequency coupling has in fact been used as a test to differentiate between the mechanisms underlying speech tracking. Oganian et al. (2023) found that when listening to slowed natural speech, a peak in IEPC was present post syllable onset at the mean syllabic rate (1.9 Hz) alongside a peak in the Theta band. By modeling this with a linear evoked model and a phase-resetting oscillator, they demonstrated that only the evoked model could reproduce this dual-peak, attributing the low frequency IEPC peak to the syllabic rate, and the high frequency peak to the frequency content of the evoked response itself. Here, we inspected the IEPC of the fast NMM’s response to our stimuli to explore the high frequency nested oscillations further. We found that the fast NMM’s IEPC has features in common with the dual spectral peak observed by Oganian et al. (2023): strong entrainment at the stimulus frequency (4 Hz), and transient entrainment at a higher frequency. This qualitatively demonstrates that an alternate mechanism could be responsible for this feature. Namely, a nested high frequency oscillation that is transiently phase-reset by syllable onsets. And further, that this can be provided natively by a mean field approximation of a biophysical network of spiking neurons; the NMM. The NMM’s response may also address recent concerns about the compatibility of generative oscillatory mechanisms with temporal response functions derived from the continuous envelope (Lalor and Nidiffer, 2025). Our results demonstrate how a nonlinear oscillatory process can respond to sharp onsets in the continuous envelope, producing nested oscillations that are free to play out between onsets.

The aim of exploring the use of the NMM is to see if we can obtain more biophysical understanding of the mechanisms responsible for speech processing in the brain. As mentioned above, an interesting biophysical fact about speech tracking is that activity in the STG represents peak-rate events rather than the continuous envelope of the speech (Oganian and Chang, 2019). We suggest that the NMM provides a mechanistic bridge between the physiology and these observations. The NMM’s activity represents the behavior of a large population of spiking neurons. We have demonstrated that when driven by the continuous envelope, these neurons effectively detect sharp onsets in the envelope, phase-locking to them. Further, it is doing so while demonstrating experimentally validated hallmarks of this processing, such as the dual-peak IEPC signature and sharpness-specific tuning of the entrainment. The peak-rate event features identified in STG activity may therefore be the result of nonlinear dynamics such as those of the NMM.

While these results provide a mechanistic candidate for modeling neural speech tracking, our investigation has several limitations. Most notably, we only qualitatively matched the dual-peak IEPC result from Oganian et al. (2023). The peaks observed in MEG occurred specifically at the stimulus rate and in the Theta band. We instead have a peak at high-beta/low-gamma, at around 30 Hz. This is a significant difference given the functionally distinct roles of theta and beta/gamma rhythms. Indeed, beta oscillations are typically suppressed during speech production and motor tasks. However, this discrepancy is due to the parameters of the fast NMM being derived to model motor beta-rhythms (Byrne et al., 2017). This parameter set was used to explore whether the NMM would entrain when its un-perturbed frequency was distinct from the 4 Hz syllabic rate. The emergence of this qualitative match to the dual-peak IEPC feature is notable, but these parameters should be tuned to determine whether a quantitative match is achievable. In addition, our test used the near-isochronous CV-syllable stimuli from the experiment by Cucu et al. (2022). This is a known confound when trying to evaluate oscillatory and evoked tracking (Lalor and Nidiffer, 2025) that partially obscured the transient phase-locking response to phoneme onsets in the IEPC test with persistent entrainment to the stimulus rate both pre- and post-onset. Further, our exploration of the simulation noise level and drive strength was quite coarse. Sensitivity analysis at a finer resolution could explore how robust the functional operating point is.

There are many opportunities for further computational investigations to confirm these results and to explore whether the NMM can support other features of neural speech processing. For instance, the NMM’s cross-frequency coupling could be tested using natural, temporally jittered speech to isolate the transient phase-resetting behavior without rhythmic interference. As was carried out in Oganian et al. (2023), the wide distribution of inter-syllable timings in natural speech could also be used to investigate the absence of sustained syllabic rate entrainment more rigorously than was possible through our inspection of the end of our periodic stimulus. Further, the cross-frequency coupling could be explored to see if it may correspond to the proposed hierarchical oscillatory structure suggested by Giraud and Poeppel (2012). Multi-region architectures may also be considered, potentially offering a way to capture the balance between bottom-up sensory processing and top-down attentional modulation in speech processing (Poeppel and Assaneo, 2020). They may also produce a better background noise representation than the stimulus-based noise used here. Adding stochasticity to the dynamics beyond the noise added to the stimulus and post hoc 1/*f* noise could result in more realistic results. Further study of the phase-reset and evoked response models could use filter-bank based models to represent tonotopic brain structures, alongside interrogation of other parameter sensitivities such as the time scale of the evoked response model. The biological plausibility of these results could be strengthened by exploring parameter ranges that are restricted to experimentally validated ranges. Subsequent tuning of these parameters could result in a closer match to experimental results. For example, tuning the synaptic timescales or membrane time constants of the NMM may shift the high IEPC peak down toward the theta range.

To confirm the biophysical mechanisms proposed by these models, targeted experimental work is required. For example, the divergence of particular phoneme groups from the experimental trend order suggests that spectral content may play a role in the final entrainment observed. An experiment following the same procedure as Cucu et al. (2022)’s with the phonemes distorted to maintain constant sharpness across conditions may reveal the effect of the spectral content of the sounds. Additionally, the correlation of phoneme sharpness with syllabic rate tracking could be investigated under natural speech conditions. This would also enable an evaluation of the NMM’s transient IEPC response to natural speech.

## Synthesis

Reviewing Editor: Anne Keitel, University of Dundee Decisions are customarily a result of the Reviewing Editor and the peer reviewers coming together and discussing their recommendations until a consensus is reached. When revisions are invited, a fact-based synthesis statement explaining their decision and outlining what is needed to prepare a revision will be listed below. The following reviewer(s) agreed to reveal their identity: Yulia Oganian, Christoph Daube. Note: If this manuscript was transferred from JNeurosci and a decision was made to accept the manuscript without peer review, a synthesis may not be available.

The manuscript "Next-Generation Neural Mass Models Reproduce Features of Speech Processing" has been re-reviewed by the two original reviewers. Both would like to thank the authors for their thorough revision and acknowledged meaningful improvements from the previous manuscript. However, there are some additional concerns, for example about the NNM being fundamentally incomparable to the oscillatory/evoked response models, and several clarification requests. The decision was therefore "Revise and resubmit".

Please respond to each comment below in a point-by-point manner.

Overall assessments - no need to respond to these

(1) The manuscript shows that a biophysical mean field model can produce activity patterns previously found in EEG and so far best explained by an evoked neural activity model. I thank the authors for addressing my previous comments. The new version of the manuscript is much easier to follow!

(2) I think the revised manuscript has several interesting datapoints contributing to the literature

- tests of PCM metric and ITPC across syllable speeds for these models

- shows NMM model shows some classically "evoked" features and some classically "oscillatory" features

- NMM is oscillatory model, but can exhibit "two peaks" of Oganian &Chang

The authors have done a great job in answering the points I raised in the first round of reviews. I think this has substantially strengthened the manuscript.

Specific comments - please respond to these

General points

1. Most generally, it seems that the NNM is a very different type of model than the oscillatory/evoked response models. While the NNM is a biophysically inspired model of meso-level network dynamics, the oscillatory/ER models are algorithmic, without claims re the underlying biophysical processes.

Thus, the NNM doesn't seem to be an alternative to these two models. Rather, my understanding is that it can be used to generating previously observed neural behavior using fixed-frequency neural oscillators. This point needs to be further qualified in the introduction. Moreover, I would still argue that the peakEnv event should be taken out/moved to the supplements. even without it, there are multiple model comparisons that a reader needs to keep track of.

The additional tests and model comparisons have improved the manuscript by a lot. but I think that there are still pieces missing that are necessary to claim that the NNM is a viable model for both - observed neural behavior - and evoked activity in the STG.

2. Reviewing this revision could have been made substantially easier if a file with intercalated figures and text had been provided. Here, figures, main text and captions are in three different locations, making it very cumbersome to read. I would like to kindly ask the editors to request authors to provide integrated manuscripts to make the lives of reviewers easier.

(Comment from the editor: Although eNeuro requires legends and figures separately, I agree that including them in the manuscript where they should appear (in addition to separate uploads), makes it much easier for reviewers to assess the work. Can I please ask to additionally integrate figures and legends into the manuscript?)

Abstract

3. "There are two competing hypotheses for the mechanisms that enable this 'neural speech tracking' - phase-resetting and evoked responses - however, we still lack understanding of the underlying cortical circuits. "

- this is written I think a bit confusingly. Easiest fix would be to slice off "however, ..." bit into a separate sentence. If there are two competing hypotheses, then the follwing bit should say something like "and these two are still unresolved".

4. "we recreate in-silico an EEG experiment identifying a correlation between tracking strength and phoneme sharpness. We compare the neural mass model to phenomenological models of the competing hypotheses and investigate their dynamics by computing the Phase Concentration Metric, "

- This is also written in a confusing way that fails to communicate the much clearer structure of the actual manuscript and its experiments. I believe the authors are really shooting in their own feet here and could win a much larger readership (which they totally deserve, I think!) by splitting this again. In my view, the point that the neural mass models of competing hypotheses are considered here should not be directly linked to the following PCM bit, but instead to the bit ending in line 48, as it directly pertains to the setup of the entire manuscript that is used for all following experiments. Then the authors should say they study this with 4 (?) different tests / experiments: The phoneme sharpness correlation as empirically demonstrated in EEG, the PCM test (here done with sine waves (?)), the syllable rate test, and the test of IEPC across phoneme onsets.

Introduction

5. line 218: 'syllabic rate test' - what this refers to is not clear without further explanation

6. "This behaviour qualitatively matches another speech tracking feature that previously has only been recreated with linear evoked models (Oganian et al., 2023)" - which one? Please be explicit

7. line 221: "the NNM facilitates speech tracking" - why facilitates? What is the primary mechanism that is facilitated by the NNM?

8. "in the activity of the auditory cortex driven by continuous speech envelopes." - is this an assumption or a result of this work?

if this is an assumption, I would disagree - there is quite some debate re what aspects of the envelope drive auditory cortex, expecially whether the continuous envelope or discrete landmarks drive it, including in work that is prominently cited in the introduction. In that sense, this conclusion would be circular.

9. syllable segregation: envelope tracking has been proposed as a mechanism for syllable segration, but whether this is the case is highly disputed, and - for my understanding - irrelevant for this project. I suggest that the authors stick to neural responses to the speech envelope/speech envelope tracking without referring to its possible algorithmic functions.

10. "This is an example of a broader debate in sensory neuroscience (Capilla et al., 2011; Zoefel et al., 2018b)."

- authors could also cite preprint by Ed Lalor and Nidiffer, which seems highly relevant to the manuscript at hand: https://osf.io/preprints/osf/xf8ay_v1

11. "For instance, starting from microscopic neuronal dynamics and including parameters that directly correspond to biological features."

- not a full sentence, please rephrase

Methods:

12. The descriptions of the models are very technical and probably not easy to understand for the average neuroscience/speech science target audience; for my part, I had a hard time understanding the NNM from this description. I suggest that the authors include a figure with schematics of all three models, their potential outcomes and opposing predictions.

13. If I understand correctly, oscillatory dynamics are built into the NNM? To what extend is it then surprising that it also shows oscillatory dynamics at the output?

14. How does the evoked response shape using for ER model compare to experimentally observer evoked responses to the envelope/peakRate events?

15. Stimulus description: thank you for all the changes, this part is now clear!

16. Why was the peakRate and peakEnv input not also used for the NNM and oscillator models?

Results:

17. Line 1014: "The two acoustic-feature impulse driven cases entrain to a lesser extent than the derivative driven case."

Is this difference significant? it appears to me that the critical question is which of the models + model settings most closely reproduce the experimental data, rather than their relative strength.

18. PCM test: so the NNM looks more similar to the evoked response model, but what do neural data look like?

19. IEPC test: the fast NNM produces a double peak at different syllables rates, that is a qualitative reproduction of the results in Oganian et al 2023. However the frequency of this high peak (25 Hz, beta range) is much higher than in that previous paper (around 8Hz). This seems an important difference: 25Hz is in the beta range, an oscillatory range with its own functional role and sources. During speech, Beta oscillations are suppressed not enhanced, as recoded with M/EEG. How do the authors explain this difference? The discussion addresses this but stops shortly of discussing the qualitative difference in frequency ranges involved, which I believe to be critical for this model comparison.would it even be possible to match the frequency ranges observed experimentally?

20. Why not use a different stimulus to drive the models, e.g., any type of natural speech?

21. "The evoked model does not respond in the same way to the envelope-stimulus."

- not sure if I have said this before, but I would call this "stimulus envelope" rather than "envelope stimulus".

22. "Using these as a baseline example of possible behaviour, we can see that both the slow and fast NMM's look most similar to the evoked model."

- could note here that for all models, there seems to be a systematic relationship between frequency and phase lag? Just for some, it has more variance, while for others, the concentration is higher? So in that sense, it is not a binary distinction as Doelling et al. could be interpreted to assert?

23. "When this model is set such that it oscillates at the syllabic rate, it successfully entrains across all rates, showing high R2 across all rates."

- but it drops at >17 Hz ?

24. "Interestingly and in contrast to the PCM experiment,"

- but also in PCM experiment, they differ? d) is narrower than c) in distribution in PCM figure?

25. Figures 7/8

- is it fair to say that if one was to plot the variance across trials of the bottom line ("amplitude"), this would start decreasing already before the onset? Why is that? Should the lines not diverge right until the onset?

Discussion:

26. Based on your results, would you discard the tuned oscillator as candidate mechanism?

27. Line 1330: "slightly more robust untuned phase resetting oscillator" please quantify and specify; as there were no untuned phase-resetting oscillators in the model comparisons.

28. It is not clear to me , where the cross-frequency coupling comes from, nor even whether this is the correct term to describe the model's dynamics. Cross-frequency coupling is typically used as a term in context of connectivity, but there is no connectivity involved here. E.g. Oganian et al argue that the two frequency peaks arise from simple additive properties of signals in the frequency domain.

Please elaborate. Similarly for the term 'gating by the slow syllable rate'.

29. My understanding of the discussion is that the NNM is proposed as a biophysical model of mechanisms underlying evoked neural activity in the STG to peakrate/acoustic edge events. I think the introduction would benefit from a clearer framing around this.

30. What about the other hallmark behaviour observed in Oganian et al, namely transient phase locking and absence of sustained phase-locking to the syllable rate? Would it be possible to evaluate that in the NNM as well? would it be feasible to add it to the manuscript?

31. Typo: "A possible explanation for of this might be the cross-frequency coupling between the slow syllabic rate and the fast intrinsic rhythm that is visible in fig. 7."

-> for of, one can go

## Author Response

Peer Review Response - Next-Generation Neural Mass Models Reproduce Features of Speech Processing We thank the editor and reviewers for their constructive and supportive reviews, which we believe have enabled us to greatly improve the quality of the manuscript. We are pleased that they found the paper relevant to the evoked-versus-oscillator debate in neural speech tracking research, and in particular that they found our model selection valuable. Along with more detailed comments, they raised two main concerns: first, that our central result did not differentiate between the models to inform on the evoked vs phase-resetting debate, and second, that we had not investigated the behaviour of the neural mass model (NMM) sufficiently. To address these, we have extended the manuscript with new tests that explore the dynamics of the NMM and how they compare to the phenomenological models' behaviour. We detail these, alongside various other smaller changes, below.

Summary of Primary Revisions 1. Phase Concentration Metric (PCM): To calculate the phase-lag between stimulus and model responses, we used the PCM; this test differentiated between evoked and phase-resetting dynamics and so offered insight into the mechanism of the NMM.

2. Syllabic Rate Variation: To further compare the behaviour of the NMM and the phenomenological models in a way that tests how well they adapt to a key property of the stimulus, we tested the effect of varying syllabic rate. This quantified the model's robustness to deviation from the 4Hz syllabic rate.

3. Inter Event Phase Coherence (IEPC): To identify whether the NMM reproduces a transient phase-locking signature associated with linear evoked responses (Oganian et al., 2023), we computed the IEPC across phoneme onsets.

Through the PCM test, we found that the phase lag between the NMM and stimulus shifts with changing stimulus rate. This indicates an evoked-like response to narrowband stimuli, sine waves in this case (Figure 1). In contrast, the syllabic rate test demonstrated that when driven by phonemes containing broadband frequency content and sharp onsets, the NMM's response was similar to a broadly tuned phase-resetting oscillator (Figure 2). We inspected the mean response of the NMM to the first phoneme and identified that it is indeed being phase-reset by the onset (Figures 3 and 4). Further investigation of the fast NMM's response to the phoneme onsets, by calculating the IEPC, revealed that, alongside phase-resetting, the onset triggered a high frequency transient oscillation that was nested within the syllabic rate. This creates two peaks in the IEPC, one at the syllabic rate, and a second transient high frequency peak after phoneme onset (Figure 5). This qualitatively matches a signature previously only attributable to linear evoked responses (Oganian et al., 2023).

Overall, these new results have given us a much more detailed description of how the NMM responds to rhythmic speech stimuli. They also clarify how the biophysically grounded dynamics of the NMM relate to the phenomenological models. By going beyond the original reproduction of the correlation between sharpness and tracking strength, we have been able to explore the NMM's dynamics; this revealed that the NMM qualitatively reproduces a further feature of speech tracking, the dual-peak IEPC signature. The NMM is performing thresholded phase-resetting to sharp onsets that trigger nested cross-frequency oscillations, effectively extracting acoustic edges from the continuous speech envelope. This indicates that the NMM's dynamics offer a biophysical explanation for the presence of peak-rate event representations in the auditory cortex. Our revised manuscript now provides a more compelling demonstration of how next-generation biophysical NMMs can bridge between physiology and cognitive computation.

We have incorporated these new results and revised the manuscript to address all of the reviewers' concerns. We present a point-by-point response to each comment below, and have submitted a tracked-changes version of the manuscript. In the tracked manuscript, new text is in bold font and deleted text has been completely removed.

1 a b c d Figure 1. Phase Concentration Vectors for each model in the PCM test. a the evoked model. b a tuned phase-resetting oscillator. c the slow NMM. d the fast NMM. Each vector, represented by a dot, is the mean phase-lag across a single response to the stimulus, at a frequency defined by the colour. The phase-resetting oscillator, and NMM's were driven by sine waves. The evoked model was driven by unit impulses aligned to the peaks of the same sine waves. Evoked responses result in a shifting phase lag as stimulus frequency changes. Phase-resetting results in a more consistent phase lag. The Phase Concentration Metric, the magnitude of the circular mean of the phase concentration vectors, is indicated by the black arrows.

Figure 2. Mean ITPC, R2 , across all phoneme condition items, as the syllabic rate is varied. Blue line, a phase-resetting oscillator tuned so that its own natural frequency matches the syllabic rate, Red line, a phase-resetting oscillator with 4Hz natural frequency. Green line, the evoked model. Purple line, the slow NMM. Gold line, the fast NMM. Shaded regions represent one standard deviation. a b Figure 3. Response of the slow NMM to the initial phoneme onset over 20 trials of the 'b' phoneme condition. Despite having random phases at the onset of the stimulus, the trajectories align due to phase resetting. Noise-to-stimulus ratio λ = 0.0 for clarity. a b Figure 4. Response of the fast NMM to the initial phoneme onset over 20 trials of the 'b' phoneme condition. Despite having random phases at the onset of the stimulus, the trajectories align due to phase resetting. Noise-to-stimulus ratio λ = 0.0 for clarity. a b Figure 5. IEPC spectrograms showing transient phase-locking response to phoneme onsets. a: Inter event phase coherence over all phoneme onsets of the "b" condition. b: IEPC minus the mean IEPC in the time range -0.25 to -0.15. There is strong 4Hz (and its harmonics) coherence across all phonemes due to the periodic nature of the stimulus. In addition to this, there is high phase coherence at around 30Hz caused by the phase reset fast oscillations. The baseline-removed plot illustrates that this is transient and locked to the onset of the phoneme.

Point by Point Response Conceptual points R1: I am unsure if the main claim (as highlighted in the title and abstract) of a clear advantage of the NMM model are really warranted. Can we really say that there is no fine-tuning of this model if we need to isolate a specific regime of parameter settings (D and lambda) to make it work well? Can the authors make it clearer that their test is just not very informative to compare the models at hand, as they all can be made to exhibit the phenomenon in question? We agree with the reviewer that our initial test did not offer sufficient mechanistic information to definitively compare the models, or to suggest a clear advantage for the NMM. We have reframed our recreation of this result as a benchmark test and explicitly state its insufficiency to distinguish between the mechanisms in the introduction, see lines 207-243.

Also, the revised first paragraph of the discussion reiterates this, see lines 1271-1276.

We have subsequently used this limitation as motivation for the new extended tests investigating the dynamics of the NMM and have made this clear in the second paragraph of the revised discussion, see lines 1277-1292.

Regarding the concern around fine tuning, we agree that the D and λ parameters required adjustment. However, we would like to make a distinction between tuning these parameters, that define the external, experimental operating conditions - input gain and background noise level respectively - and those that determine the model behaviour. By setting D and λ we were effectively identifying a map between the conditions the models were exposed to and those of the experiment, finding that the correlation arose at the point where the mean entrainment strength matched that observed through EEG. Beyond testing the two distinct variations of the NMM, the slow and fast settings, we explicitly did not tune the internal model parameters. Furthermore, we added equivalent environmental tuning for the two phenomenological models, for instance hard-coding the drive amplitude through normalisation for the phase-resetting oscillator, and sweeping λ for both of them. To ensure this is made clear, we have added the following lines in the methods section: 656-663.

R2: It is not entirely clear what the crucial aspect of the NMM model is that leads to it exhibiting the phenomenon in question. Could one therefore argue that "one black box is replaced with another"? Can the authors interpret their findings in some way that makes it clear in what specific way the NMM brings about the differential reactions to sharpness? A2: We agree that it would have been beneficial in the original manuscript to provide a mechanistic insight into the behaviour of the NMM. While it is grounded in the biophysical details of the underlying network of spiking neurons it is derived from, we accept that this does not inherently mean that its macroscopic dynamics are interpretable. We particularly appreciate this request for further investigation as this was the focus of our extensions to the manuscript. As detailed in the Summary of Revisions above, the new results demonstrate that the NMM is performing thresholded phase resetting to phoneme on- sets triggering high frequency nested oscillation. This allows it to extract acoustic edges from the continuous envelope and may be what facilitates the differential reaction to sharpness. We hope the new results provide the mechanistic detail sought here.

R3: Lines 791 - 805: The authors argue that a model that can reproduce the neural results based on the continuous envelope is more parsimonious. I would agree with this argument if nothing was known about cortical envelope processing and representation. However, as acoustic edge representations have been demonstrated in auditory speech cortex, unlike envelope representations (by Oganian &Chang, 2019, which the authors also cite on multiple occasions), this argument here doesn't make sense, as the authors themselves somewhat argue in the next paragraph. Likewise, given the same set of results, modeling results based on envelope peak input cannot be used to argue about a model's plausibility as no evidence exists for a cortical representation of envelope peaks.

A3: We agree that our original framing, that processing the continuous envelope was more parsimonious, contradicted the established result that acoustic edge representations rather than envelope representations are present in auditory speech cortex. We have completely re-written this section of the discussion and we explicitly highlight this point when we discuss the difference in the model's required stimuli, see lines 1261-1271.

Further, this comment motivated our subsequent investigation into the NMM's behaviour. The NMM's ability to reproduce the sharpness-correlation directly from the continuous envelope did offer some indication that it was attending to peak-rate events, as phoneme sharpness is directly linked to the peak-rate timing and magnitude. This was confirmed in our dynamical investigations that showed that the NMM was indeed performing edge extraction from the continuous envelope, therefore offering an explanation for how the acoustic-edge representations might arise in auditory cortex. This is something that the evoked model assumed a-priori through its use of pre-processed peak-rate impulses.

Regarding the peak-envelope impulses, we agree that they are not biologically plausible features, but have opted to retain them as a baseline. We have made this explicit in the text, see lines 528-536 in the methods.

R4: Lines 845 - 846: as prior work has already shown that the oscillatory model fails to account for other aspects of speech tracking for non-isochronous more ecologically valid inputs (e.g., Oganian, Kojima et al 2023), the authors should make clear that their argument is about neural responses to an isochronous acoustic input, not general mechanisms of speech perception A4: We agree that our results are limited by the use of the near-isochronous rhythmic input from Cucu et al. (2022)'s experiment and that this should be clear throughout the manuscript. While our new results indicate that the behaviour of the NMM may not rely on the rhythmicity of the input, instead containing a transient response to the phoneme onsets that suggests the new results may transfer to natural speech, we have revised the manuscript to make the use of, and limitation of, the isochronous stimuli clear in the discussion. See lines 1242, 1449-1456, and 1468 − 1473.

R5: As is, the key contribution of this work remains unclear and not well integrated with the prior literature.

A5: We agree that our original focus on the recreation of the sharpness correlation obscured our primary contribution and that it appeared insufficiently integrated with prior findings. However, with the new dynamical investigations into the NMM's response, the manuscript no longer presents only the phenomenological fit to the correlation. We now demonstrate the production of a further macroscopic feature of neural speech processing, the transient high frequency response to phoneme onsets (as seen in the IEPC). Further, we offer an explanation for how acoustic edge representations in auditory cortex could arise from networks of neurons processing the continuous speech envelope. Ultimately, this serves as a validation of the NMM itself as a model of generic cortical dynamics. To make our contributions clear, we have substantially revised the introduction, highlighting our intent to explore biophysical modelling of speech processing rather than to just contribute to the phase-resetting vs evoked debate, see lines 178-206. We have also highlighted the contribution of our new analyses and their potential implication at the end of the introduction, see lines 243-265.

In addition, we have carefully considered the additional works provided by the reviewers and have integrated them into the manuscript. See the Additional proposed citations section, A27 below for details.

R6: 106 - 110: If there is envelope tracking, it necessarily only refers to energy fluctuations, which goes against the initial argument of "syllables are often not separated by pauses". I read "pauses" in the latter argument as "energy fluctuations" (i.e. silence vs signal). So envelope tracking doesn't solve the problem of how the brain projects syllables onto sustained energy.

A6: Thank you for pointing out the potential ambiguity in our phrasing. We respectfully disagree, however, with the point that envelope tracking requires pauses, or that speech lacks trackable energy fluctuations. When stating that syllables are often not separated by pauses, our intention was to convey that there are rarely periods of silence between them. Indeed, this aligns with the reviewer's point that speech consists of sustained energy. However, sustained energy, or a lack of pauses, does not rule out amplitude modulation. Therefore, while a lack of discrete silent gaps between syllables makes segregation difficult, the brain can use these above-zero fluctuations to track the envelope. To ensure this is clear we have modified the second sentence in the introduction, see lines 119-122.

R7: line 234: "entrainment" would make a strong commitment to a model where endogenous oscillations are present in the neural response and then adjust their frequency to track the stimulus. As far as I am aware, this is a matter of debate for auditory stimuli (to which this manuscript contributes). A more neutral "tracking" might be a good choice at this point in the manuscript? A7: Thank you for highlighting this, we agree that entrainment does favour the oscillatory hypothesis and have updated the manuscript to use tracking instead where appropriate.

R8: Line 644: Can the authors provide a brief high-level description here how it would be theoretically possible for the oscillator model to react to input without the phase reset mechanism? This would make the claim that the phase-reset mechanism is necessary more compelling.

A8: Overall, the intent with the modulated phase-resetting oscillator was to investigate how much the particular instantiation of phase-resetting in the model - the sinusoidal modulation of the input according to the phase at which it was applied - by slowly reducing how strong this modulation was. When we removed the phase resetting mechanism, we were not completely removing the impact of the stimulus, only this sinusoidal modulation by the phase. So it would be possible for the model to react to the stimulus without phase resetting, however this would only be in a single direction, i.e an impulse could only advance the phase of the model. Other mechanisms for entrainment such as synchronisation could have been present however yes, this would be expected to remove the entrainment effect. We appreciate that this may not have been clear in the text, and have removed both our modulation of the phase-resetting strength and the natural frequency sweep to avoid confusion. These have in some part been replaced by the PCM, and syllabic rate tests later on.

Methodological/structural points R9: Lines 821 on: could the authors add the analyses proposed here to the manuscript? A9: Thank you for this prompt which motivated the main part of our revisions. We have added extensive new analyses to investigate the NMM's dynamics as described above in the Summary of Revisions. In particular we focused on further empirical tests to allow comparison to experimental results (for instance Oganian et al., 2023, and Doelling et al., 2019). This included the PCM test as proposed in the prior version of the manuscript, as well as a test of the effect of changing syllabic rate and an evaluation of the IEPC across phoneme onsets. We hope these address the concern raised here.

Regarding our original suggestion to compute the phase response curve (PRC), we realized upon further investigation that calculating a formal PRC requires the assumption that the model remains on a particular stable limit cycle. Given the cross- frequency transient behaviour of the NMM demonstrated in the new IEPC test, this assumption wouldn't hold for the NMM. During the transient response, the model moves onto a different cycle, which will not necessarily have the same phase response characteristics as the baseline steady-state cycle. Accordingly, we have removed this suggestion from the revised discussion.

R10. Overall, if the aim of this project was to introduce NMMs as a potential alternative mechanism for modeling speech tracking, in particular tracking of the amplitude envelope of the speech signal, it would be critical to reproduce other known phenomena related to speech tracking. Especially so, as the correlation between envelope sharpness and ITPC and evoked response magnitude have been reported in the past (Oganian et al 2023, but see also prior work in auditory electrophysiology in animal models and in humans, e.g. work by Peter Heil and Israel Nelken).

A10: We agree that fully establishing NMMs as a viable alternative for modelling speech tracking requires more than the reproduction of the correlation in our original manuscript. This was a primary focus of our revision, and we thank the reviewers for suggesting this as an avenue for improvement.

As detailed in the Summary of Revisions section, we have now characterised the NMM's dynamics through the PCM, syllabic rate, and IEPC tests. In doing so, we have demonstrated that the NMM qualitatively recreates the transient high frequency phase-locking to phoneme onsets observed by Oganian et al,. (2023). Specifically, the NMM is phase-reset by the phoneme onsets and also produces high frequency oscillations nested within the syllabic rhythm. The NMM is effectively performing acoustic edge extraction through this process, allowing it to track the amplitude envelope of the speech signal. This provides fur- ther evidence of the NMM's potential for successfully capturing the link between biophysical circuit dynamics and macroscale cognitive computations.

R11. My key takeaway is that sharpness-related changes in ITPC cannot distinguish between the different models at hand. Thus to introduce NMM as a viable alternative to the other models, a deeper analyses of its properties and fit to other aspects of speech tracking would be necessary.

A11: We agree with the reviewers concern: that the correlation of tracking strength with sharpness appears to be a degenerate feature for distinguishing between these models.

We aimed to resolve this with our new tests. As detailed in the Summary of Revisions above, and in A10, we investigated the model's dynamics, with a focus on the NMM. We used the PCM to identify the model's response to simple stimuli, followed by the syllabic rate test to expose any differences caused by syllable-based drive of varying rates. Finally, we demonstrated with the IEPC test that the NMM qualitatively reproduced another experimentally verified feature of speech tracking: the dual-peak IEPC result from Oganian et al., 2023.

These new results successfully distinguish the NMM from the phenomenological models:

1. All three models can produce the sharpness correlation with tracking strength.

2. Only the NMM and evoked model demonstrate robustness to syllabic rate variations within the natural range for speech.

3. Only the NMM or evoked models (Oganian et al., 2023) produce the dual-peaked IEPC response to phoneme onsets 4. Only the NMM provides an explanation of how populations of neurons could transform continuous acoustic envelope inputs into peak-rate representations through acoustic edge extraction.

R12. Line 216: "tests" is an unusual word. "items" seems the default nomenclature in psycholinguistic / neurolinguistic research. This seems like a favourable choice not least to avoid confusion with the "tests" mentioned in line 283.

A12: Agreed, thank you for pointing this out. We have updated the experiment description, and elsewhere, to refer to items instead of tests. For an example, see lines 462 and 502.

R13: line 221: More details about the noise are needed, as well as about the signal-to-noise ratio. Also, does this describe adding noise to the audio signal? As in, "to simulate recording the neural response", we now have > 16kHz signals with noise? Figure 1 caption states that envelopes were extracted before adding noise, which would make somewhat more sense. This information seems to be provided at a later stage, but so the order needs to be changed to be less confusing for the reader A13: Thank you for highlighting that this section was difficult to follow. Indeed, the noise was added after the envelopes were extracted. To make this more clear, we have removed this particular description of the trials and noise addition (it was not in an ideal place) and moved it into a dedicated subsection on the creation of our stimuli. Overall, the methods section has been significantly reordered and revised to improve flow and understanding. See lines 468-553.

R14: line 225: "mean ITPC over the three test" at this point I have no idea where the "three" comes from. 15 conditions, each with 60 trials for each of 45 items? It says earlier that there are "3 distinct auditory streams corresponding to different vowels in the CV pairs", but then in 218, we see 4 different vowels. So here I am lost.

A14: We apologise for the convoluted test description, the section of the methods describing this experiment has been heavily revised, and re-ordered to ensure that this information is clearly described. There are three stimulus streams for each condition, the "items" as in A12, that have different random vowel sequences (each using all of the possible vowels, hence the four in the example item.). Each one had 60 different instances of noise added to it to make 60 trials per item. Alongside the change from 'tests' to 'items' (see A12), our revisions will hopefully make it more understandable. See the new "Experimental Stimuli" and "Stimulus Construction" sections of the methods, lines 450-553.

R15: Line 251: "We used "latency to maximum derivative", L, to measure phoneme sharpness" - since this appears to be a central measure for this project, please make it much more explicit how this is measured. E.g., specify with respect to what the latency was defined.

A15: Thank you for identifying this lack of explanation, we have now explicitly stated the how the latency was calculated in Cucu et al (2022)'s paper. See lines 571-584.

R16: line 299: What is the "phase reset mechanism"? How the model implements this is not transparent at this point. The details seem to follow later, but so wouldn't it be a good idea to describe these details first and then refer to them assuming they are known? A16: Agreed, the details did come after this, so we have updated the structure of the methods so that the model descriptions now come first. In any case, we have also removed the modulation of the phase-resetting from the manuscript in accordance with R8/A8 above, so this particular case is no longer an issue.

R17: line 363: "of 20 CV phonemes" - aren't these CV syllables? Or phoneme pairs? This section seems to describe what is also picked up on in lines 220 - 230. I am not sure why this appears in two disjoint sections of the methods. A17: Thank you for pointing out this discrepancy. The line referred to has been removed to avoid the duplicate description of the stimuli, and all other references to the CV-syllables as phonemes has been corrected. The methods section has also been restructured to improve comprehension. Specifically, there is now a distinct section for the CV-syllable experiment, where we first introduce the EEG experiment's stimuli, followed by describing how we convert these into stimuli for our simulation, then proceeding onto finer details of simulation settings and post-hoc noise. See lines 443-467 and 468-553.

R18: Line 635 / Table 3 / Figures 3,4: We need the correlation value obtained from EEG here for comparison with the values obtained from the models. I would even suggest to move table 3 to the supplemental material and instead provide a figure that has mean and some variance estimate around mean (e.g. SEM or std) of EEG and all models. Likewise, figure 2 and 3 could be merged into a single figure with EEG and all models. It is not clear to me why NMM and phase reset models are plotted without, but evoked models are plotted with direct comparison to EEG results. Can this be made more coherent? A18: We agree that figure 2 and 3 should be combined as they are presenting the same result for the different models. We have done so, and retained the first subplot for the EEG result, this avoids clutter on the subplots and makes it a six panel figure. Accordingly we have removed the EEG results from the evoked response subplots. We have also included the EEG correlation, along with its 95% confidence interval and p-value in Table 3.

R19: Further, it seems that there are 2 quantities of interest that one would want to match to data: The correlation of ITPC over latency to maximum derivative as well as the absolute values of ITPC. Would it make sense to use a different metric that would integrate these two quantities into one? E.g. coefficient of determination. This could be evaluated directly against the (average) EEG data. This would both be sensitive to the strength of the relationship over L as well as the absolute values of ITPC. With that, one could make plots showing this coefficient of determination as a function of parameters such as lambda and D, giving a heatmap for each model. This would condense the somewhat complex figure S2 (with 8 subpanels) into 2 subpanels, which would be interesting for the main text, and keep figure S3 to 8 subpanels. A19: Yes, thank you for pointing this out, we had considered this issue but opted for just the mean ITPC as it meant there would be no ambiguity about what was causing a match or disagreement between the model values and the EEG-based results. The suggestion that we could condense the information in figures S2 and S3 is appreciated however. So, we have replaced figures figures 4 and 5 in the paper, (showing the change in mean ITPC with drive strength and noise strength) with figure 6. This presents a heatmap of the concordance correlation coefficient over noise vs drive strength for the NMM, and noise vs model type for the others. The concordance correlation coefficient evaluates both the correlation and scale agreement of the data, giving a value between 0 and 1 (a perfect match). This naturally obscures what causes a low score as both a different correlation, and a different scale can result in a low score. To ensure that the raw data underlying the score remains accessible, we have retained figures S2 and S3 in the supplementary material. This also means that the individual ITPCs per phoneme are still presented somewhere for all conditions. a b c Figure 6. Heatmaps of the concordance correlation coefficient, ρ , between the mean ITPC, R2 , per phoneme condition of the models and the EEG data. a, b: the slow and fast NMM respectively, as D and λ are varied. c: for each model as λ is varied. The admissible range of ρ values is between 0.0 and 1.0, with 1.0 indicating a perfect match between the models R2 values and those obtained from the EEG cucu2022syllable. The full, per-phoneme-condition, ITPCs for each model under every experimental condition are presented in fig. 3-1 and fig. 3-2.

R20: line 655: "with the derivative stimulus" - isn't it less confusing to call it "the derivative of the stimulus envelope"? Calling it "derivative stimulus" makes it seem like this is a different stimulus presented to man and machine, but as I understand, it is just a different feature space extracted from the same stimulus which is fed into the evoked model here. A20: Agreed, you are correct that it is just a feature extracted from the stimulus envelope, thank you for highlighting that this was not clear. We have updated the text accordingly, see line 588.

R21: Line 867 following: This somewhat reads a bit like one would have to manipulate the NMM to exhibit the sharpness dependent responses? Or is it meant as a description of a process that must be happening "inside" of the NMM? A21: Thank you for pointing out the ambiguity in this paragraph. The original intent was to describe the underlying mechanisms that may be occurring in the NMM, however as we now have new results that directly interrogate this mechanism, we have removed this paragraph entirely, replacing it with discussion of the new findings. See new discussion on lines 1277-1402.

Minor points R22: color maps in the figures are not easily seen, I suggest improving legend readability and maybe color and sizes A22: Thank you for bringing this to our attention, we have updated the figures legends to make them clearer.

R23: Table 3: add correlations in original EEG data set please A23: See response A18. Experimental EEG correlations added to Table 3, including 95% confidence intervals and p-value.

R24: line 267 (and other places): "Hochburg" -> Hochberg A24: Thank you for bringing this to our attention! We have corrected all instances of this mistake.

R25: Table 1: I think the colour highlighting is fantastic and I urge the journal to adopt this in the final publication. (Editor note: Not sure this can be influenced, but I agree that it is useful colour-coding) A25: Thank you for the kind feedback, it is good to hear that the colour coding was useful. We appreciate that it may not be possible to preserve in the final formatting though.

R26: line 907: "tonotopic style brain structures" sounds a bit colloquial? Simply removing the "style" should help making it sound more professional? Same for "filter bank style" - could say "filter bank based"? A26: Agreed, we have updated the text as suggested, see lines 1494-1495.

Additional proposed citations R27: Line 112: Could cite work of Okko Räsänen, Cognition 2018 Line 123 - 125: Could cite Hertrich et al. 2012 here.

Line 118: Could cite Capilla et al., 2011; as well as Zoefel et al., frontiers 2018 Line 129: Could cite Zou et al., eNeuro 2021 Line 142: Doelling et al. 2019 is not about speech processing, but tones.

Line 159: Could cite Daube et al., 2019 / Oganian et al 2023 also show this for speech syllables A27: Thank you for highlighting these relevant papers. Including them has provided stronger context for our contribution both in terms of experimental findings and computational investigations. Specifically we have integrated them into the text as follows: • Räsänen et al., 2018: Included to support our statement that speech tracking is a mechanism of syllable segregation, line 126. • Hertrich et al., 2012: Included as more evidence for discrete onset features presence in neural activity, line 145. • Capilla et al., 2011; Zoefel et al., 2018: Used to frame the evoked vs phase-resetting debate in speech processing as being part of the larger discussion in sensory research, lines 137-138. • Zou et al., 2021: Included as a further instance of mathematical models supporting the evoked hypotheses, combined with Oganian et al., 2023 into one remark about their capture of features in 'macroscale neural recordings' to account for them using EEG and MEG data respectively, line 150. • Doelling et al., 2019: Thank you for catching this, we have removed this particular instance of this citation. • Daube et al., 2019/Oganian et al., 2023: We have included these citations as further evidence for the significance of the sharpness-tuning observed by Cucu et al., 2022 in a new paragraph that frames our use of their data as a benchmark. See lines 227-229.

Further revisions:

Various typographical errors have been corrected. To adhere to the word limit, some sentences have been cut/adjusted.

References . Oganian Y, Kojima K, Breska A, Cai C, Findlay A, Chang EF, Nagarajan SS (2023) Phase alignment of low-frequency neural activity to the amplitude envelope of speech reflects evoked responses to acoustic edges, not oscillatory entrainment. Journal of Neuroscience 43:3909-3921.
