# Supplementary material for: Next-Generation Neural Mass Models Reproduce Features of Speech Processing
Source: eNeuro. 2026 Aug 21;13(8):ENEURO.0379-25.2026. doi: 10.1523/ENEURO.0379-25.2026 (PMC13505880; doi:10.1523/ENEURO.0379-25.2026)
Supplement: Supplementary file 1 [file eneuro-13-ENEURO.0379-25.2026-s006.docx]

| Model | $\lambda$ | $D$ | $r$ | $\text{CI}_{\text{lower}}$ | $\text{CI}_{\text{upper}}$ | $p$ | $p_{\text{Bonferroni}}$ | $p_{\text{FDR}}$ |
| --- | --- | --- | --- | --- | --- | --- | --- | --- |
| NMM fast | 0 | 0.1 | -0.852 | -0.95 | -0.604 | 5.417e-05 | 0.000433 | 0.000157 |
| NMM fast | 0 | 0.496 | 0.269 | -0.282 | 0.687 | 0.332 | 1 | 0.379 |
| NMM fast | 0 | 2.46 | 0.66 | 0.222 | 0.876 | 0.00748 | 0.0598 | 0.0199 |
| NMM fast | 0 | 12.2 | -0.585 | -0.844 | -0.104 | 0.022 | 0.176 | 0.0293 |
| NMM fast | 0 | 60.5 | 0.632 | 0.177 | 0.865 | 0.0114 | 0.0916 | 0.0183 |
| NMM fast | 0 | 100 | 0.764 | 0.414 | 0.917 | 0.000912 | 0.0073 | 0.00243 |
| NMM fast | 0.05 | 0.1 | -0.477 | -0.795 | 0.0469 | 0.0723 | 0.579 | 0.116 |
| NMM fast | 0.05 | 0.496 | 0.129 | -0.41 | 0.602 | 0.647 | 1 | 0.647 |
| NMM fast | 0.05 | 2.46 | 0.437 | -0.097 | 0.776 | 0.103 | 0.827 | 0.207 |
| NMM fast | 0.05 | 12.2 | -0.144 | -0.611 | 0.397 | 0.608 | 1 | 0.608 |
| NMM fast | 0.05 | 60.5 | 0.517 | 0.00638 | 0.814 | 0.0485 | 0.388 | 0.0969 |
| NMM fast | 0.05 | 100 | 0.575 | 0.089 | 0.84 | 0.0249 | 0.199 | 0.0665 |
| NMM fast | 0.1 | 0.1 | -0.852 | -0.95 | -0.604 | 5.422e-05 | 0.000434 | 0.000175 |
| NMM fast | 0.1 | 0.496 | 0.177 | -0.369 | 0.632 | 0.529 | 1 | 0.529 |
| NMM fast | 0.1 | 2.46 | 0.832 | 0.557 | 0.942 | 0.000121 | 0.000966 | 0.000322 |
| NMM fast | 0.1 | 12.2 | -0.588 | -0.845 | -0.108 | 0.0212 | 0.17 | 0.0283 |
| NMM fast | 0.1 | 60.5 | 0.439 | -0.0943 | 0.777 | 0.101 | 0.812 | 0.116 |
| NMM fast | 0.1 | 100 | 0.725 | 0.339 | 0.902 | 0.00221 | 0.0177 | 0.00441 |
| NMM fast | 0.3 | 0.1 | -0.844 | -0.947 | -0.586 | 7.472e-05 | 0.000598 | 0.000299 |
| NMM fast | 0.3 | 0.496 | 0.0161 | -0.5 | 0.524 | 0.954 | 1 | 0.954 |
| NMM fast | 0.3 | 2.46 | 0.828 | 0.549 | 0.941 | 0.000136 | 0.00109 | 0.000545 |
| NMM fast | 0.3 | 12.2 | -0.479 | -0.796 | 0.0441 | 0.0709 | 0.567 | 0.113 |
| NMM fast | 0.3 | 60.5 | -0.0262 | -0.531 | 0.493 | 0.926 | 1 | 0.926 |
| NMM fast | 0.3 | 100 | 0.39 | -0.152 | 0.752 | 0.15 | 1 | 0.172 |
| NMM fast | 0.5 | 0.1 | -0.818 | -0.937 | -0.526 | 0.000194 | 0.00155 | 0.000777 |
| NMM fast | 0.5 | 0.496 | -0.186 | -0.637 | 0.361 | 0.508 | 1 | 0.58 |
| NMM fast | 0.5 | 2.46 | 0.488 | -0.0322 | 0.8 | 0.0649 | 0.519 | 0.13 |
| NMM fast | 0.5 | 12.2 | -0.517 | -0.814 | -0.0064 | 0.0485 | 0.388 | 0.0969 |
| NMM fast | 0.5 | 60.5 | -0.634 | -0.865 | -0.18 | 0.0112 | 0.0896 | 0.0179 |
| NMM fast | 0.5 | 100 | -0.522 | -0.816 | -0.0135 | 0.0459 | 0.367 | 0.0917 |
| NMM fast | 0.7 | 0.1 | -0.751 | -0.912 | -0.388 | 0.00125 | 0.00997 | 0.00499 |
| NMM fast | 0.7 | 0.496 | -0.329 | -0.72 | 0.221 | 0.231 | 1 | 0.617 |
| NMM fast | 0.7 | 2.46 | -0.0897 | -0.576 | 0.443 | 0.751 | 1 | 0.751 |
| NMM fast | 0.7 | 12.2 | 0.147 | -0.395 | 0.613 | 0.602 | 1 | 0.705 |
| NMM fast | 0.7 | 60.5 | -0.178 | -0.633 | 0.368 | 0.525 | 1 | 0.7 |
| NMM fast | 0.7 | 100 | -0.477 | -0.795 | 0.0471 | 0.0724 | 0.579 | 0.193 |
| NMM fast | 0.9 | 0.1 | -0.57 | -0.838 | -0.0814 | 0.0265 | 0.212 | 0.0354 |
| NMM fast | 0.9 | 0.496 | -0.306 | -0.707 | 0.245 | 0.267 | 1 | 0.318 |
| NMM fast | 0.9 | 2.46 | -0.709 | -0.896 | -0.309 | 0.00307 | 0.0245 | 0.00491 |
| NMM fast | 0.9 | 12.2 | -0.909 | -0.97 | -0.742 | 2.716e-06 | 2.173e-05 | 2.173e-05 |
| NMM fast | 0.9 | 60.5 | 0.767 | 0.42 | 0.918 | 0.000844 | 0.00675 | 0.00113 |
| NMM fast | 0.9 | 100 | 0.564 | 0.0729 | 0.835 | 0.0285 | 0.228 | 0.0456 |
| NMM fast | 1 | 0.1 | -0.338 | -0.725 | 0.211 | 0.218 | 1 | 0.34 |
| NMM fast | 1 | 0.496 | -0.28 | -0.693 | 0.271 | 0.312 | 1 | 0.357 |
| NMM fast | 1 | 2.46 | -0.156 | -0.619 | 0.387 | 0.579 | 1 | 0.579 |
| NMM fast | 1 | 12.2 | -0.183 | -0.636 | 0.363 | 0.514 | 1 | 0.514 |
| NMM fast | 1 | 60.5 | 0.509 | -0.00474 | 0.81 | 0.0528 | 0.422 | 0.249 |
| NMM fast | 1 | 100 | 0.208 | -0.34 | 0.651 | 0.456 | 1 | 0.456 |
