# Supplementary material for: Next-Generation Neural Mass Models Reproduce Features of Speech Processing
Source: eNeuro. 2026 Aug 21;13(8):ENEURO.0379-25.2026. doi: 10.1523/ENEURO.0379-25.2026 (PMC13505880; doi:10.1523/ENEURO.0379-25.2026)
Supplement: Supplementary file 2 [file eneuro-13-ENEURO.0379-25.2026-s007.docx]

| Model | $\lambda$ | $D$ | $r$ | $\text{CI}_{\text{lower}}$ | $\text{CI}_{\text{upper}}$ | $p$ | $p_{\text{Bonferroni}}$ | $p_{\text{FDR}}$ |
| --- | --- | --- | --- | --- | --- | --- | --- | --- |
| NMM slow | 0 | 0.1 | 0.85 | 0.599 | 0.949 | 5.893e-05 | 0.000471 | 0.000157 |
| NMM slow | 0 | 0.496 | 0.162 | -0.382 | 0.623 | 0.563 | 1 | 0.563 |
| NMM slow | 0 | 2.46 | 0.285 | -0.266 | 0.695 | 0.304 | 1 | 0.304 |
| NMM slow | 0 | 12.2 | -0.617 | -0.858 | -0.153 | 0.0143 | 0.115 | 0.0229 |
| NMM slow | 0 | 60.5 | 0.733 | 0.353 | 0.905 | 0.00189 | 0.0151 | 0.00503 |
| NMM slow | 0 | 100 | 0.345 | -0.203 | 0.729 | 0.208 | 1 | 0.208 |
| NMM slow | 0.05 | 0.1 | 0.843 | 0.581 | 0.946 | 8.050e-05 | 0.000644 | 0.000322 |
| NMM slow | 0.05 | 0.496 | 0.173 | -0.373 | 0.629 | 0.539 | 1 | 0.615 |
| NMM slow | 0.05 | 2.46 | 0.261 | -0.29 | 0.682 | 0.347 | 1 | 0.347 |
| NMM slow | 0.05 | 12.2 | -0.634 | -0.865 | -0.181 | 0.0111 | 0.0888 | 0.0296 |
| NMM slow | 0.05 | 60.5 | 0.767 | 0.42 | 0.918 | 0.000845 | 0.00676 | 0.00225 |
| NMM slow | 0.05 | 100 | 0.493 | -0.0255 | 0.803 | 0.0618 | 0.494 | 0.102 |
| NMM slow | 0.1 | 0.1 | 0.835 | 0.563 | 0.944 | 0.000108 | 0.000865 | 0.000216 |
| NMM slow | 0.1 | 0.496 | 0.201 | -0.347 | 0.646 | 0.473 | 1 | 0.529 |
| NMM slow | 0.1 | 2.46 | 0.224 | -0.325 | 0.661 | 0.421 | 1 | 0.421 |
| NMM slow | 0.1 | 12.2 | -0.637 | -0.867 | -0.186 | 0.0106 | 0.0848 | 0.017 |
| NMM slow | 0.1 | 60.5 | 0.801 | 0.489 | 0.931 | 0.000334 | 0.00267 | 0.00089 |
| NMM slow | 0.1 | 100 | 0.598 | 0.124 | 0.85 | 0.0185 | 0.148 | 0.0246 |
| NMM slow | 0.3 | 0.1 | 0.802 | 0.493 | 0.932 | 0.000318 | 0.00254 | 0.000536 |
| NMM slow | 0.3 | 0.496 | 0.343 | -0.205 | 0.727 | 0.211 | 1 | 0.281 |
| NMM slow | 0.3 | 2.46 | 0.253 | -0.298 | 0.677 | 0.363 | 1 | 0.369 |
| NMM slow | 0.3 | 12.2 | -0.0636 | -0.558 | 0.464 | 0.822 | 1 | 0.822 |
| NMM slow | 0.3 | 60.5 | 0.792 | 0.47 | 0.928 | 0.000436 | 0.00349 | 0.000871 |
| NMM slow | 0.3 | 100 | 0.482 | -0.0405 | 0.797 | 0.069 | 0.552 | 0.11 |
| NMM slow | 0.5 | 0.1 | 0.765 | 0.416 | 0.918 | 0.000892 | 0.00713 | 0.00143 |
| NMM slow | 0.5 | 0.496 | 0.26 | -0.291 | 0.682 | 0.349 | 1 | 0.49 |
| NMM slow | 0.5 | 2.46 | 0.111 | -0.425 | 0.59 | 0.693 | 1 | 0.693 |
| NMM slow | 0.5 | 12.2 | 0.464 | -0.0639 | 0.789 | 0.0818 | 0.655 | 0.131 |
| NMM slow | 0.5 | 60.5 | 0.721 | 0.332 | 0.901 | 0.0024 | 0.0192 | 0.00479 |
| NMM slow | 0.5 | 100 | -0.162 | -0.623 | 0.382 | 0.563 | 1 | 0.644 |
| NMM slow | 0.7 | 0.1 | 0.71 | 0.31 | 0.896 | 0.00305 | 0.0244 | 0.00813 |
| NMM slow | 0.7 | 0.496 | -0.0153 | -0.523 | 0.501 | 0.957 | 1 | 0.957 |
| NMM slow | 0.7 | 2.46 | -0.538 | -0.823 | -0.0349 | 0.0387 | 0.31 | 0.103 |
| NMM slow | 0.7 | 12.2 | -0.69 | -0.888 | -0.274 | 0.00444 | 0.0355 | 0.0178 |
| NMM slow | 0.7 | 60.5 | 0.352 | -0.195 | 0.732 | 0.198 | 1 | 0.528 |
| NMM slow | 0.7 | 100 | 0.235 | -0.315 | 0.667 | 0.399 | 1 | 0.614 |
| NMM slow | 0.9 | 0.1 | 0.573 | 0.0855 | 0.839 | 0.0257 | 0.205 | 0.0354 |
| NMM slow | 0.9 | 0.496 | 0.328 | -0.222 | 0.719 | 0.233 | 1 | 0.318 |
| NMM slow | 0.9 | 2.46 | -0.567 | -0.836 | -0.0768 | 0.0276 | 0.221 | 0.0368 |
| NMM slow | 0.9 | 12.2 | -0.854 | -0.95 | -0.606 | 5.180e-05 | 0.000414 | 0.000104 |
| NMM slow | 0.9 | 60.5 | -0.768 | -0.919 | -0.422 | 0.000817 | 0.00654 | 0.00113 |
| NMM slow | 0.9 | 100 | 0.533 | 0.0281 | 0.821 | 0.0409 | 0.327 | 0.0545 |
| NMM slow | 1 | 0.1 | 0.357 | -0.19 | 0.735 | 0.191 | 1 | 0.34 |
| NMM slow | 1 | 0.496 | 0.456 | -0.0729 | 0.785 | 0.0873 | 0.698 | 0.249 |
| NMM slow | 1 | 2.46 | 0.424 | -0.113 | 0.769 | 0.115 | 0.924 | 0.249 |
| NMM slow | 1 | 12.2 | -0.37 | -0.742 | 0.176 | 0.175 | 1 | 0.35 |
| NMM slow | 1 | 60.5 | -0.256 | -0.679 | 0.295 | 0.358 | 1 | 0.409 |
| NMM slow | 1 | 100 | 0.396 | -0.146 | 0.755 | 0.144 | 1 | 0.288 |
