# Supplementary material for: Next-Generation Neural Mass Models Reproduce Features of Speech Processing
Source: eNeuro. 2026 Aug 21;13(8):ENEURO.0379-25.2026. doi: 10.1523/ENEURO.0379-25.2026 (PMC13505880; doi:10.1523/ENEURO.0379-25.2026)
Supplement: Supplementary file 3 [file eneuro-13-ENEURO.0379-25.2026-s008.docx]

| Model | $\lambda$ | $r$ | $\text{CI}_{\text{lower}}$ | $\text{CI}_{\text{upper}}$ | $p$ | $p_{\text{Bonferroni}}$ | $p_{\text{FDR}}$ |
| --- | --- | --- | --- | --- | --- | --- | --- |
| Evoked derivative | 0 | -0.617 | -0.858 | -0.153 | 0.0142 | 0.114 | 0.019 |
| Evoked derivative | 0.05 | -0.49 | -0.801 | 0.0301 | 0.0639 | 0.511 | 0.073 |
| Evoked derivative | 0.1 | -0.303 | -0.706 | 0.248 | 0.272 | 1 | 0.272 |
| Evoked derivative | 0.3 | 0.25 | -0.301 | 0.676 | 0.369 | 1 | 0.369 |
| Evoked derivative | 0.5 | 0.251 | -0.3 | 0.676 | 0.367 | 1 | 0.42 |
| Evoked derivative | 0.7 | -0.107 | -0.587 | 0.429 | 0.705 | 1 | 0.705 |
| Evoked derivative | 0.9 | -0.879 | -0.959 | -0.667 | 1.596e-05 | 0.000128 | 0.000102 |
| Evoked derivative | 1 | -0.45 | -0.782 | 0.0815 | 0.0927 | 0.742 | 0.332 |
| Evoked envelope | 0 | 0.87 | 0.644 | 0.956 | 2.542e-05 | 0.000203 | 0.000157 |
| Evoked envelope | 0.05 | 0.864 | 0.632 | 0.954 | 3.236e-05 | 0.000259 | 0.000213 |
| Evoked envelope | 0.1 | 0.862 | 0.627 | 0.953 | 3.572e-05 | 0.000286 | 0.000175 |
| Evoked envelope | 0.3 | 0.899 | 0.716 | 0.966 | 5.237e-06 | 4.190e-05 | 4.190e-05 |
| Evoked envelope | 0.5 | 0.79 | 0.466 | 0.927 | 0.000459 | 0.00367 | 0.00122 |
| Evoked envelope | 0.7 | 0.559 | 0.0661 | 0.833 | 0.0301 | 0.241 | 0.0603 |
| Evoked envelope | 0.9 | -0.303 | -0.706 | 0.248 | 0.273 | 1 | 0.312 |
| Evoked envelope | 1 | 0.205 | -0.344 | 0.649 | 0.464 | 1 | 0.464 |
| Evoked peak-env | 0 | 0.792 | 0.47 | 0.928 | 0.000438 | 0.0035 | 0.000876 |
| Evoked peak-env | 0.05 | 0.809 | 0.506 | 0.934 | 0.000261 | 0.00209 | 0.000522 |
| Evoked peak-env | 0.1 | 0.848 | 0.593 | 0.948 | 6.569e-05 | 0.000526 | 0.000175 |
| Evoked peak-env | 0.3 | 0.807 | 0.502 | 0.933 | 0.00028 | 0.00224 | 0.000536 |
| Evoked peak-env | 0.5 | 0.769 | 0.423 | 0.919 | 0.000816 | 0.00653 | 0.00143 |
| Evoked peak-env | 0.7 | -0.235 | -0.667 | 0.315 | 0.399 | 1 | 0.614 |
| Evoked peak-env | 0.9 | -0.824 | -0.94 | -0.539 | 0.000159 | 0.00127 | 0.000423 |
| Evoked peak-env | 1 | 0.421 | -0.116 | 0.768 | 0.118 | 0.942 | 0.332 |
| Evoked peak-rate | 0 | -0.382 | -0.748 | 0.162 | 0.161 | 1 | 0.161 |
| Evoked peak-rate | 0.05 | -0.4 | -0.757 | 0.141 | 0.139 | 1 | 0.139 |
| Evoked peak-rate | 0.1 | -0.467 | -0.79 | 0.0591 | 0.079 | 0.632 | 0.0903 |
| Evoked peak-rate | 0.3 | -0.443 | -0.779 | 0.0895 | 0.0981 | 0.785 | 0.112 |
| Evoked peak-rate | 0.5 | -0.111 | -0.59 | 0.425 | 0.693 | 1 | 0.693 |
| Evoked peak-rate | 0.7 | -0.207 | -0.65 | 0.342 | 0.46 | 1 | 0.614 |
| Evoked peak-rate | 0.9 | -0.87 | -0.956 | -0.644 | 2.540e-05 | 0.000203 | 0.000102 |
| Evoked peak-rate | 1 | -0.414 | -0.764 | 0.124 | 0.125 | 0.996 | 0.332 |
