# Supplementary material for: Next-Generation Neural Mass Models Reproduce Features of Speech Processing
Source: eNeuro. 2026 Aug 21;13(8):ENEURO.0379-25.2026. doi: 10.1523/ENEURO.0379-25.2026 (PMC13505880; doi:10.1523/ENEURO.0379-25.2026)
Supplement: Supplementary file 4 [file eneuro-13-ENEURO.0379-25.2026-s009.docx]

| Model | $\lambda$ | $r$ | $\text{CI}_{\text{lower}}$ | $\text{CI}_{\text{upper}}$ | $p$ | $p_{\text{Bonferroni}}$ | $p_{\text{FDR}}$ |
| --- | --- | --- | --- | --- | --- | --- | --- |
| No phase reset | 0 | 0.634 | 0.18 | 0.865 | 0.0112 | 0.0897 | 0.0179 |
| No phase reset | 0.05 | 0.675 | 0.249 | 0.882 | 0.00572 | 0.0458 | 0.00916 |
| No phase reset | 0.1 | 0.726 | 0.34 | 0.902 | 0.0022 | 0.0176 | 0.00351 |
| No phase reset | 0.3 | 0.513 | 0.0012 | 0.812 | 0.0504 | 0.403 | 0.0672 |
| No phase reset | 0.5 | 0.357 | -0.19 | 0.735 | 0.192 | 1 | 0.256 |
| No phase reset | 0.7 | 0.128 | -0.412 | 0.601 | 0.65 | 1 | 0.705 |
| No phase reset | 0.9 | 0.165 | -0.379 | 0.625 | 0.557 | 1 | 0.557 |
| No phase reset | 1 | -0.3 | -0.704 | 0.251 | 0.277 | 1 | 0.34 |
| Phase reset | 0 | -0.571 | -0.838 | -0.0837 | 0.026 | 0.208 | 0.0298 |
| Phase reset | 0.05 | -0.624 | -0.861 | -0.164 | 0.013 | 0.104 | 0.0173 |
| Phase reset | 0.1 | -0.658 | -0.875 | -0.219 | 0.00771 | 0.0617 | 0.0103 |
| Phase reset | 0.3 | -0.801 | -0.931 | -0.489 | 0.000335 | 0.00268 | 0.000536 |
| Phase reset | 0.5 | -0.865 | -0.954 | -0.634 | 3.099e-05 | 0.000248 | 0.000248 |
| Phase reset | 0.7 | -0.892 | -0.964 | -0.699 | 7.880e-06 | 6.304e-05 | 6.304e-05 |
| Phase reset | 0.9 | -0.785 | -0.925 | -0.457 | 0.00052 | 0.00416 | 0.00104 |
| Phase reset | 1 | 0.288 | -0.263 | 0.697 | 0.298 | 1 | 0.34 |
